# Comparison of pre-processing methods for multiplex bead-based immunoassays

**DOI:** 10.1186/s12864-016-2888-7

**Published:** 2016-08-11

**Authors:** Tanja K. Rausch, Arne Schillert, Andreas Ziegler, Angelika Lüking, Hans-Dieter Zucht, Peter Schulz-Knappe

**Affiliations:** 1 Institut für Medizinische Biometrie und Statistik, Universität zu Lübeck, Universitätsklinikum Schleswig-Holstein, Campus Lübeck, Ratzeburger Allee 160, 23562 Lübeck, Germany; 2 Zentrum für Klinische Studien, Universität zu Lübeck, Lübeck, Germany; 3 School of Mathematics, Statistics and Computer Science, University of KwaZulu-Natal, Pietermaritzburg, South Africa; 4 Protagen AG, Dortmund, Germany

**Keywords:** Autoantibody, Bead-based, Immunoassay, Luminex, Multiplex, Omics, Pre-processing, Protein

## Abstract

**Background:**

High throughput protein expression studies can be performed using bead-based protein immunoassays, such as the Luminex® xMAP® technology. Technical variability is inherent to these experiments and may lead to systematic bias and reduced power. To reduce technical variability, data pre-processing is performed. However, no recommendations exist for the pre-processing of Luminex® xMAP® data.

**Results:**

We compared 37 different data pre-processing combinations of transformation and normalization methods in 42 samples on 384 analytes obtained from a multiplex immunoassay based on the Luminex® xMAP® technology. We evaluated the performance of each pre-processing approach with 6 different performance criteria. Three performance criteria were plots. All plots were evaluated by 15 independent and blinded readers. Four different combinations of transformation and normalization methods performed well as pre-processing procedure for this bead-based protein immunoassay.

**Conclusions:**

The following combinations of transformation and normalization were suitable for pre-processing Luminex® xMAP® data in this study: weighted Box-Cox followed by quantile or robust spline normalization (rsn), asinh transformation followed by loess normalization and Box-Cox followed by rsn.

**Electronic supplementary material:**

The online version of this article (doi:10.1186/s12864-016-2888-7) contains supplementary material, which is available to authorized users.

## Background

Bead-based protein immunoassays using the Luminex® xMAP® technology are subject to variability caused by both biological and technical effects. While systematic effects resulting from differences in biological conditions are of interest, technical variability should be reduced to the minimum. The highest proportion of technical variability is systematic and potentially introduced during different protein processing steps [1, 2].

In the ideal experimental setting, all samples would be processed in a single run, and, depending on the aim of the study, all analytes would be measured simultaneously or each analyte separately. However, technical limitations do not permit such an approach.

Bead-based immunoassays are a technological derivative to conventional immunoassays such as ELISAs, where antigen/antibody reactions are measured. The solid phase of the ELISA plate is reduced to multiple, small fluorescent color-coded bead particles, which allows the conduction of multiplex experiments by simultaneous incubation of different bead species with samples. The analytical readouts are fluorescence signals reading each bead color (attribute channel) together with the signal from fluorescence labeled antibodies or proteins (quantitative measure).

Currently, 500 different bead colors can be differentiated, allowing for the simultaneous analysis of 500 analytes with the Luminex® xMAP® technology. Furthermore, well-plate layouts and robotic automation requirements typically restrict the number of used samples per batch to 96 or 384. In consequence, any large-scale analysis needs to be run in batches, which can introduce technical variability on the sample level and the analyte level [3].

The presence of technical variability generally affects downstream statistical analysis. For example, the power for detecting biological effects may be reduced or effect estimates may be biased. As a result, the reduction of technical effects is mandatory for reliable protein expression analysis, and a suitable pre-processing strategy is required for minimizing technical variability.

The most important steps for data pre-processing are transformation and normalization of raw data after initial quality control [4]. The optimal pre-processing approach should be carefully selected prior to data analysis based on both the employed technology and the actual data [5] because the pre-processing method may greatly influence downstream analysis. As a result, microarray gene expression data are pre-processed differently [6] than RT-PCR data [7] or data from genotype microarrays [8, 9]. Several authors compared methods to find optimal techniques for data pre-processing of different Omics-type-of data [5, 10–14]. However, pre-processing methods have not been compared for the Luminex® xMAP® technology.

The aim of this paper therefore is to identify an appropriate approach for the pre-processing of multiplex data generated with the Luminex® xMAP® technology. The analytical setting investigated here, is based on the reaction of the presence of human autoantibodies in patient serum to identify their binding partners. For this purpose we couple recombinantly produced human proteins to different, color coded beads and let them simultaneously react with individual serum samples. We used control sera and sera of patients having the autoimmune diseases multiple sclerosis and neuromyelitis optica for demonstration purposes. In summary, the assay is a multiplexed direct immunoassay with autoantibodies (IgG) as target analytes.

To this end, we compared 37 different combinations of transformation and normalization for a real data set of 384 analytes (i.e. antibody – antigen reactions) using 42 serum samples.

## Methods

### Biological experiment

#### Subjects

The data considered in this study consisted of 384 potential auto-antigens measured for 42 serum samples. The ethics committee of the Heinrich-Heine-Universität of Düsseldorf approved this study (vote number 2850, January 22, 2007). All participants gave written informed consent. The samples data were obtained from 12 measurements from a pooled reference serum, 12 control samples and 30 affected subjects (18 patients with multiple sclerosis, 12 patients with neuromyelitis optica). The 42 patient samples were measured on four plates. A reference serum was measured 12 times for each analyte. Additionally, 12 measurements of a pooled serum sample were measured three times on four plates each. This was used for estimating the repeatability of measurements. The amount of antibodies was measured as signal intensities using the Luminex® xMAP® technology in combination with the FLEXMAP 3D® instrument in serums of cases and controls.

#### Wet lab procedures

All 384 protein antigens were recombinantly produced in-house, using *E.coli* and a SCS1 carrying plasmid pSE111, containing an N-terminally located hexa-histidine-tag [15, 16]. Each purified antigen was coupled to magnetic carboxylated color-coded beads (MagPlexTM microspheres, Luminex Corporation, Austin, Texas). The manufacturer’s protocols were adapted to enable multiplexing using semi-automated procedures. All liquid handling steps were carried out by either an eight-channel pipetting system (Starlet, Hamilton Robotics, Bonaduz, Switzerland) or a 96-channel pipetting system (Evo Freedom 150, Tecan, Männedorf, Switzerland). For each coupling reaction up to 12.5 μg antigen and 8.8 × 10^5^ MagPlexTM beads per color were used. Finally, beads were combined and stored at 4–8 °C until use.

#### Autoantibody profiling

Serum samples were diluted 1:100 in assay buffer (PBS, 0.5 % BSA, 50 % Low-Cross buffer (Candor Biosciences, Wangen, Germany)), added to the bead mix of 384 proteins and incubated for 20 h at 4–8 °C. After washing with PBS/0.05 % Tween20 the beads were incubated with a fluorescence labeled (R-phycoerythrin) detection antibody (5 μg/ml, goat anti-human or goat-anti-mouse IgG, Dianova, Hamburg, Germany) for 45 min at RT to detect the target analyte, antigen-specific human IgG species from human serum.

The beads were washed and then analyzed in a FlexMap3D instrument (Luminex Corporation, Austin, Texas). The instrument aspirates the beads containing patient antibodies bound to the respective protein antigens, and which have bound the detection antibody, and analyses each individual particle by using a flow cytometric technology. The analytical measure is the median fluorescence intensity (MFI) for the particles partitioned according to their respective identification color. According to the manufactures recommendations, the MFI readout fulfilling a minimum bead count criterion (>35 beads measured per bead ID) were exported for data analysis.

### Pre-processing procedure

The following steps were used for data pre-processing: First, raw data were quality controlled. In brief, antigens with a proportion of null values exceeding 19 % and samples with a proportion of null values exceeding 20 % were excluded. Signal intensities ≤ 0 were set to missing values. Second, we applied a transformation to the quality-controlled data. Next, we imputed missing data by median imputation [17] to the transformed and quality controlled data. Finally, we applied a normalization method to the data.

### Transformation and normalization methods

We used the notation *transformation*_*normalization* to label the used methods for the transformation and normalization, which we applied as combinations during the pre-processing procedure to the data. Here, *transformation* is one of the following transformations: no transformation (*no*), log_2_ transformation (*log2*), asinh transformation (*asinh*), Box-Cox transformation (*boxcox*) [18], Box-Cox transformation with weights (*boxcoxweights*) [18] and variance stabilizing transformation (*vst*) [19]. boxcox is the original Box-Cox transformation, where the transformation *y*_*t*_ is obtained as ${y}_t=\frac{y^{\lambda }-1}{\lambda }$ if *λ* ≠ 0 and *y*_*t*_ = *log y* if *λ* = 0. The transformation boxcoxweights uses the geometric mean $\overset{.}{y}$ as a weight so that ${y}_t=\frac{y^{\lambda }-1}{\lambda {\overset{.}{y}}^{\lambda -1}\ }$ if *λ* ≠ 0 and ${y}_t= log\ (y)\cdot \overset{.}{y}$ if *λ* = 0.

The *normalization* method was one of the following: loess normalization (*loess*) [20], global median normalization (*global*) [21], quantile normalization (*quantile*) [22], an improved quantile normalization (*quanimpr*), robust spline normalization (*rsn*) [23], z-score normalization (*zscore*) or variance stabilizing normalization (*vsn*) [24]. vsn has a built-in transformation, and it was therefore applied directly to the quality controlled and imputed data. The improved quantile normalization is a modification of the common quantile normalization, which we have developed to deal with very few large signal intensities. Specifically, borrowing from the technique of dithering in digital video and audio signal processing [25], noise was added to the original dataset to reduce the influence of the few strong signals on the normalization.

### Evaluation criteria

We used 6 different criteria to evaluate the effects of the pre-processing methods. Two of the six criteria were based on empirical thresholds for statistical characteristics describing the distribution of the data, one measures variation of the signal intensities, and the remaining three criteria were based on visual inspection of plots. All evaluation criteria were graded as poor, fair or good and scored with 0, 1 and 2, respectively, for all pre-processing methods. Fifteen blinded readers rated the plots independently. The readers rated the plots twice i.e., at two different time points, where plots of the pre-processing methods were shuffled for the second run to test intra-rater reliability. Plots could reach a score between 0 and 30 and were classified as good (2) for a score between 21 and 30, as fair (1) for 11 to 20 and poor (0), otherwise. As total score we added the scores of the 6 criteria, and the pre-processing methods could reach a total score between 0 and 12. The best pre-processing method was the one with the highest score when the evaluation criteria were summed. The evaluation criteria used are described in detail in the following sections.

#### Mean-SD (standard deviation) plot

In Mean-SD plots ranked means are plotted against the standard deviation. If the variability, i.e., standard deviation of intensities, depends on the magnitude of measured intensities, data are not homoscedastic. This, in turn, invalidates the use of many statistical methods, such as the analysis of variance (ANOVA) [26]. The variation should therefore be independent of measured signal intensities, thus independent of the mean in an optimally pre-processed data set. We estimated the mean and the standard deviation from the reference pool serum for each pre-processing combination.

**Fig. 1:**
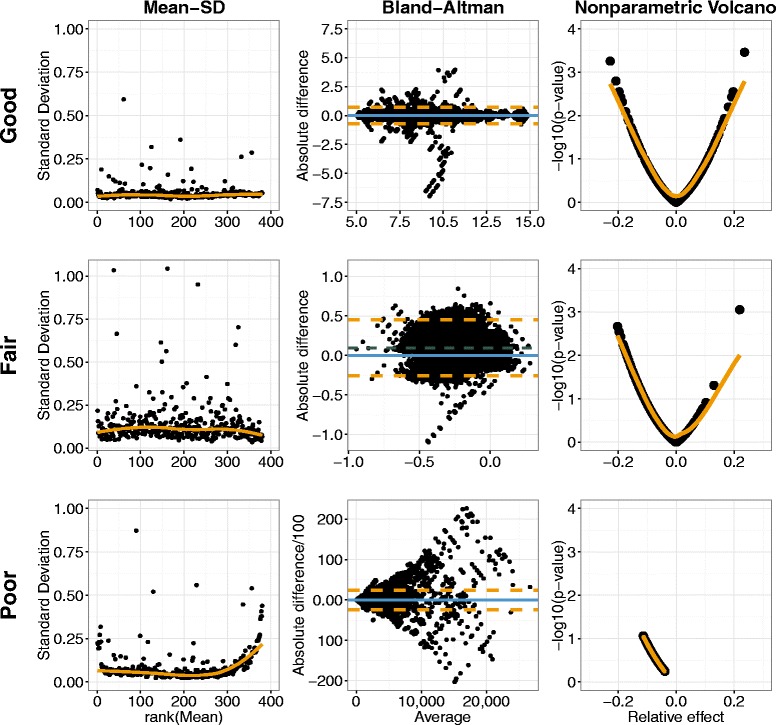
Examples for a good, a fair and a poor mean-standard deviation, Volcano and Bland-Altman plot. Mean-SD plot: *orange line*: loess curve; good: scatterplot parallel to x-axis with low variation, sd stable over mean signal intensities; fair: no trend to larger sd for larger means but variation around loess curve; poor: trend. Bland-Altman plot: *blue line*: *zero line*; *green line*: mean difference; *orange lines*: 95 % CI; good: mean of differences close to zero with small, constant variation around this mean; fair: neither trend nor funnel but mean difference deviating widely from zero or increased scatter; poor: visible trend or funnel. Volcano plot: *orange line*: loess curve; good: visible funnel, both sides with similar length; fair: one side of the funnel considerably shorter than the other; poor: no funnel shape

The rating instructions for the raters of this plot were the following: If the scatterplot parallels the x-axis with low variation and the standard deviation is stable over the mean of signal intensities, the pre-processing method has to be judged as good (2). The loess curve (orange) in the plot should help to identify a potential trend; a trend should be judged as poor (0). Plots with no trend to a larger standard deviation for larger means but a variation around the loess curve have to be judged as fair (1). Figure 1 shows the example plots, which were given to the raters to help them with their decision.

#### Bland-Altman plot

The Bland-Altman plot [27] is generally used to plot the difference of two measurements against their mean where one is a new method to find out how much the new method differs from the old one. Here, we plotted all pairs of the 12 measurements in the reference pool serum for each pre-processing method in one Bland-Altman plot. The following rating instruction was given to the blinded readers: The mean of the differences should be close to zero with a small and constant variation around this mean. If these criteria are fulfilled, the plot should be judged as good (2). A plot with a visible trend or funnel has to be judged as poor (0). If neither trend nor funnel but a mean difference deviating widely from zero or an increased scatter is present, the plot has to be judged as fair (1).

#### Volcano plot

In general, −log_10_ transformed *p*-values are plotted against log_2_ fold changes in volcano plots [28]. The *p*-values are taken from the *t*-test. In this situation *p*-values were estimated for cases versus control using the nonparametric Wilcoxon rank sum test because antigen intensities might not be normally distributed. Hence, rank-based relative effects [29] as a nonparametric effect measure were used instead of fold changes. The shape of the plot is therefore a funnel and not the typical volcano shape as relative effects and *p*-values for the Wilcoxon-test are based on the same rank sums.

The evaluation instructions for volcano plots were the following: For plots where a funnel is visible and both sides are similar in length, the plot has to be judged as good (2). If one side of the funnel is considerably shorter than the other the plot has to be judged as fair (1). If the plot has no funnel shape at all the plot has to be judged as poor (0).

#### Skewness and tail length

Skewness and tail length were determined to assess similarities to the normal distribution for the distribution of the signal intensities of all data. Both statistics are computed through quantile estimators.

Skewness was estimated by $log\ S= log\frac{{\tilde{x}}_{0.975}-{\tilde{x}}_{0.5}}{{\tilde{x}}_{0.5}-{\tilde{x}}_{0.025}}$ [30], where ${\tilde{x}}_q$ denotes the *q* -quantile. For symmetric distributions *log S* equaling zero, and it is negative or positive for left-skewed and right-skewed distributions, respectively. Tail length was estimated by $T=\frac{{\tilde{x}}_{0.975}-{\tilde{x}}_{0.025}}{{\tilde{x}}_{0.875}-{\tilde{x}}_{0.125}}$ [30], which can take values between 1 and infinity [30]. The larger *T*, the longer the tail of a distribution. The normal distribution has a tail length of *T* = 1.704.

Thresholds were taken from the literature for scoring skewness and tail length [31]. A distribution was almost symmetric if − 0.5 < *log S* < 0.5 and scored with 2. If *log S* deviated more than 0.75 from 0, it received a score of 0; otherwise it received a score of 1.

Similarly, the tail length of the distribution was judged to be good (2), i.e., close to the normal distribution, if 1.625 < *T* < 2. The score for tail length was 0 if *T* ≤ 1.525 or *T* ≥ 2.1, and otherwise it received a score of 1.

#### Coefficient of variation

We used the coefficient of variation (CV) to judge repeatability by considering the 12 measurements from the reference pool serum. A pooled measure was computed for each pre-processing method in the following steps:

1. Get CVs for each antigen in each pre-processed data set separately.
2. Rank CVs for one antigen over all pre-processed data sets; start with the smallest.
3. Sum these ranks (*CV*_*s*_) across all antigens for each pre-processing method separately.

A small value for *CV*_*s*_ indicates that this pre-processing method has small CVs for the majority of antigens; smallest possible *CV*_*s*_ equals the number of antigens, highest is the product of the number of antigens and of the pre-processing methods.

Before scoring *CV*_*s*_ it was transformed to percentages *CV*_*s*,*p*_ and scored with 2 if *CV*_*s*,*p*_ ≤ 50 %, with 1 if 50 % < *CV*_*s*,*p*_ ≤ 80 %, and 0, otherwise.

### QQ-plot

To illustrate the effects of the pre-processing we randomly drew equally sized groups from one case group and performed Mann–Whitney U tests. We repeated this 25 times and plotted it in a QQ-plot. If the pre-processing reduces variability between subjects the lines in the QQ-plot should scatter narrowly around the diagonal line.

### Software used

R version 3.1.1 was used together with Bioconductor Version 3.0 for all computations and visualizations [32]. For both Box-Cox transformations we employed the R-function boxcox (package MASS (7.3-40)) for estimating λ. Unweighted Box-Cox transformed data were obtained from the R-function BoxCox (package forecast (5.9)). The R-function bct (package TeachingDemos (2.9)) transforms data with the weighted Box-Cox transformation but cannot handle missing data. We therefore implemented this transformation as an R-function. We used the function lumiN from the Bioconductor-package lumi (2.18.0) to perform quantile normalization, loess normalization, vsn and rsn. All plots were generated using the R package ggplot2 (1.0.1).

## Results

A systematic literature search was used to identify methods for transformation and normalization (Table 1). Search criteria were combinations of “transformation”, “normalization”, “preprocess”, “comparison”, “microarray” and modifications of them. We only included methods which were already implemented in the statistical software R [33] or simple to implement. Furthermore, we aimed at investigating the effects of no transformation. In total, we applied 37 different combinations of 6 transformation methods and 7 normalization methods to the data. We excluded 4 of 384 antigens during the quality control for further studies because values were missing for at least 8 of 42 patients (19.05 %).

**Table 1:** Methods for transformation and normalization identified through literature search

Transformation		
Name	Abbr. in paper	Reference
Hyperbolic area sine*	asinh	[37]
Box-Cox*	boxcox	[11]
Log2*	log2	[5, 38]
Linlog		[12]
No***	no	-
Variance stabilizing*	vst	[5, 38]
Weighted Box-Cox**	boxcoxweights	-
Normalization		
Name	Abbr. in paper	Reference
Contrast		[10]
Cyclic loess		[10]
Global mean		[39]
Global median*	global	[39]
Housekeeping genes		[17]
Improved quantile**	quanimpr	-
Invariant set		[10]
Locally weighted scatterplot smoothing*	loess	[5]
Peng’s method		[13]
Quantile*	quantile	[5, 10, 38]
Qspline		[10, 40]
Robust quantile		[10]
Robust spline*	rsn	[5, 38]
Scaling/constant		[10]
Spiked controls		[17]
T-quantiles		[13]
Tukey’s biweight scaling		[13]
Variance stabilizing*	vsn	[5, 40]
Z-score*	zscore	[41]

Methods used in this work are marked with at least one asterisk. Own developed methods are marked with two asterisks. We have chosen the methods with three asterisks to test their effects on the data

**Fig. 2:**
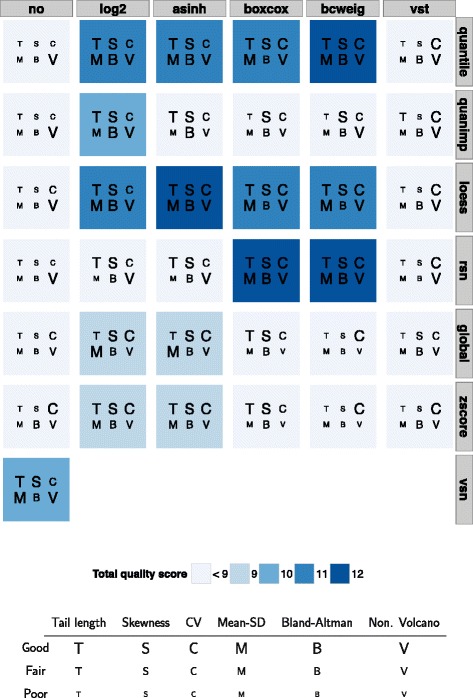
Total quality scores of the 37 different pre-processing methods over all 6 evaluation criteria. Single quality scores take the values 0 (poor, small letter), 1 (fair, medium-sized letter) and 2 (good, tall letter) per criterion. bcweig: boxcoxweights; CV: Coefficient of variation; Mean-SD: mean-standard deviation

Scores for the visual ratings and the statistical characteristics are provided in Fig. 2 for the 37 different combinations of transformation and normalization methods for 6 different evaluation criteria. Each criterion was either assessed as poor, fair or good, corresponding to the letter sizes small, medium and tall in Fig. 1. We considered the first of the two runs for the plot evaluation for the sum of scores. The following four pre-processing methods obtained the maximum total score of 12:

1. Asinh transformation with loess normalization,
2. Box-Cox transformation with robust spline normalization,
3. Weighted Box-Cox transformation with quantile normalization and
4. Weighted Box-Cox transformation with robust spline normalization.

In general, pre-processing methods without a transformation and methods with a variance stabilizing transformation (VST) reached small total scores. The improved quantile normalization reached only a higher total score in combination with the log_2_ transformation. Global median and z-score normalization had a highest total score of 9 in combination with either log_2_ transformation or asinh transformation but failed, otherwise. Figure 3 shows the QQ-plot of the raw data, and Fig. 4 the QQ-plots of the four best pre-processing methods. Additional file 1: Figure S1 shows a selection of QQ-plots with pre-processing combinations with smaller total quality scores. Finally, (Additional file 1: Figures S2–S38) shows the QQ-plots of all 37 combinations of the investigated pre-processing methods.

**Fig. 3:**
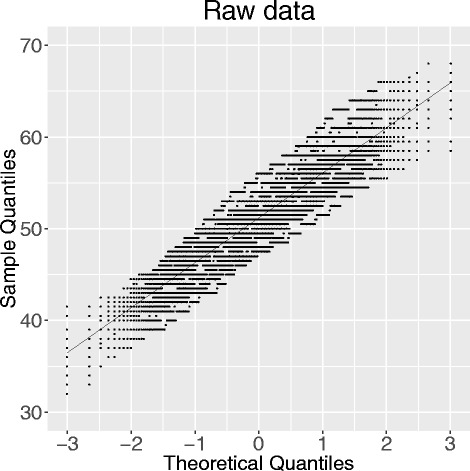
QQ-plot, raw data. QQ-plot of test statistics of the Mann–Whitney *U* test, drawn subsamples of the two case groups, 25 replications

**Fig. 4:**
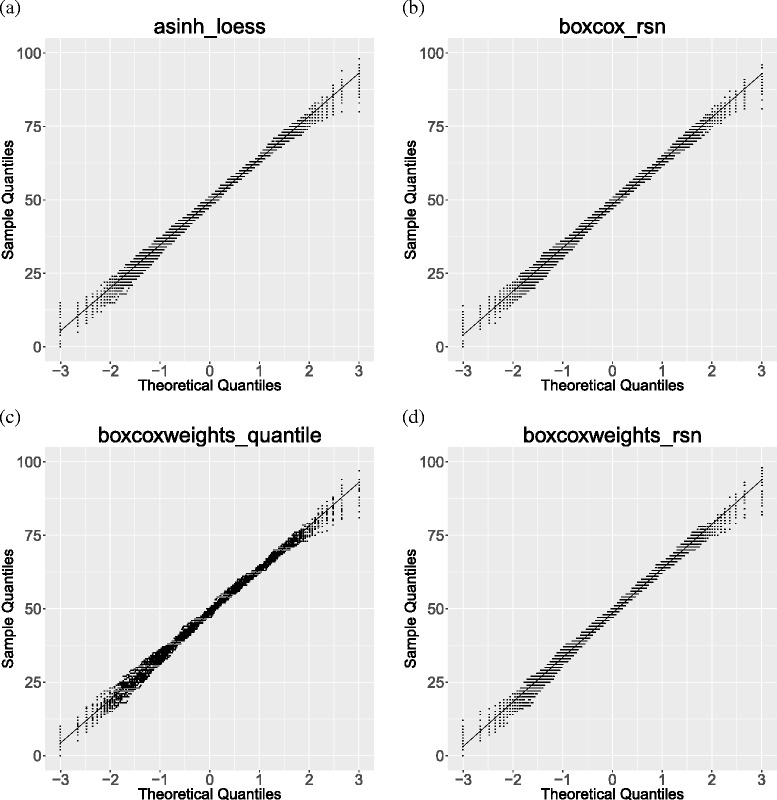
QQ-plots, four best pre-processing combinations (**a**)–(**d**). QQ-plots of test statistics of the Mann–Whitney *U* test, drawn subsamples of the two case groups, 25 replications Loess and quantile normalization performed well in combination with all transformations but no transformation and the VST. The variance stabilizing normalization reached a high total score. Additional file 2: Table S1 provides detailed results for skewness, tail length and the coefficient of variation for all pre-processing methods. Additional file 1: Figure S39 - S75 shows the resulting plots for all 37 pre-processing combinations with a Mean-SD, Bland-Altman and nonparametric Volcano plot. These plots were shown to the blinded readers. Intra-rater reliability was evaluated using the correlation coefficient for the total quality scores which was 0.99 between both rounds.

## Discussion

The best four approaches for pre-processing the Luminex® xMAP® data identified in this work were a weighted Box-Cox transformation followed by a quantile, a robust spline normalization (rsn), an asinh transformation followed by a loess normalization and a Box-Cox transformation followed by an rsn. Our findings demonstrate that data transformation is necessary prior to downstream analysis, as all combinations without prior transformation reached considerably bad evaluation scores. Unexpectedly, the VST was rated poorly in this study although this approach performed well in gene expression studies [5, 6]. In the future, it would be helpful if other groups replicated our findings using independent data. The results of the QQ-plots show how the results in one case group behave after pre-processing. The scattering of the test statistics around the line in the QQ-plot of the raw data (Fig. 3) is much larger than in the QQ-plots of the four best methods (Fig. 4). In comparison, the QQ-plots of *log2_rsn* and *vst_loess* show a larger scattering and the QQ-plots of *boxcox_global* and *boxcoxweights_zscore* scatter largely and are inflated (Additional file 1: Figure S1).

To ensure all important information are stored for proteomics experiments for further data handling, a standard reporting guideline for minimum information about a proteomics experiment (MIAPE) has been developed for methods, such as gel electrophoresis and mass spectrometry [34]. However, MIAPE standards are lacking for the Luminex® xMAP® technology. Such a development would be important for future reports of experiments based on the Luminex® xMAP® technology. At this stage our aim was to provide data handling recommendations to allow for later in-depth analysis of the different steps in laboratory work including Luminex-based data generation. To allow for this, we here produced first data sets following recommendations from Luminex both for multiplex assay setup and raw data collection.

A limitation of the transformation methods in our study is the usage of the same method for all antigens within the transformation step except for both Box-Cox transformations. If each antigen is transformed separately, results might be different. This should, however, be investigated in future studies. Another limitation of this study is the small sample size (42 samples in total). As a result, the power of group comparisons is limited. However, this sample size has been used in very early stages of several biomarker studies.

As demonstrated by Ziegler et al. [35], the coefficient of variation varies with the strength of gene expression and decreases with increasing expression levels. For that reason removal of transcripts with low intensity values from expression data with a detection call algorithm [36] is often used. In this study, we followed standard manufacturer recommendations and used data only if there were at least 35 beads. The dependency of the coefficient of variation on the number of beads warrants further investigation.

In summary, our investigation about appropriate data transformation and normalization methods for the Luminex® xMAP® technology has shown that either one of the four following data pre-processing approaches is appropriate: a weighted Box-Cox transformation followed by a robust spline normalization, an asinh transformation followed by a loess normalization, a Box-Cox transformation followed by an rsn and a weighted Box-Cox transformation followed by a quantile normalization.

## Conclusions

We identified four adequate transformation methods for antigen intensities obtained by the Luminex® xMAP® technology using simple graphical and statistical characteristics. The suitable methods are a weighted Box-Cox transformation followed by a quantile or robust spline normalization (rsn), an asinh transformation followed by a loess normalization or a Box-Cox transformation followed by an rsn.

## Additional files

Additional file 1: Supplementary figures. All results for the QQ-, Mean-SD, Bland-Altman and Volcano plots. (PDF 5133 kb)

Additional file 2: Supplementary table. Detailed results for the evaluation criteria skewness, tail length and coefficient of variation. (PDF 54 kb)

Additional file 3: Supplementary material. Datasets, which support the conclusions of this article. (ZIP 58 kb)
