# Supplementary material for: Comparison of pre-processing methods for multiplex bead-based immunoassays
Source: BMC Genomics. 2016 Aug 11;17:601. doi: 10.1186/s12864-016-2888-7 (PMC4982217; doi:10.1186/s12864-016-2888-7)
Supplement: Supplementary file 1 — Supplementary figures. All results for the QQ-, Mean-SD, Bland-Altman and Volcano plots. (PDF 5133 kb) [file 12864_2016_2888_MOESM1_ESM.pdf]

Supplement for: *Comparison of Pre-processing Methods for Multiplex Bead-Based Immunoassays*

Tanja K. Rausch, Arne Schillert, Andreas Ziegler, Angelika Lueking, Hans-Dieter Zucht, Peter Schulz-Knappe

Part 1: QQ-plots, examples for pre-processing combinations with higher scatter variation

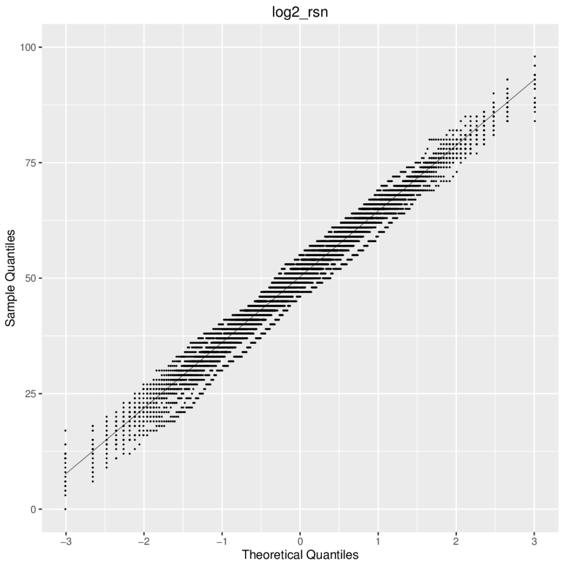

(a) Total quality scores: 8

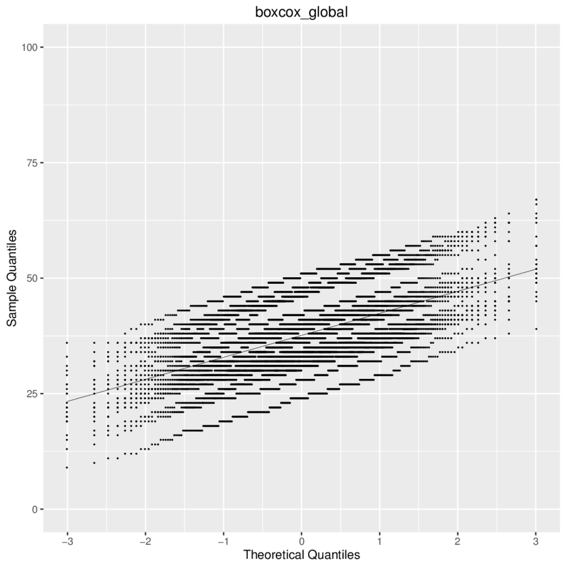

(b) Total quality score: 6

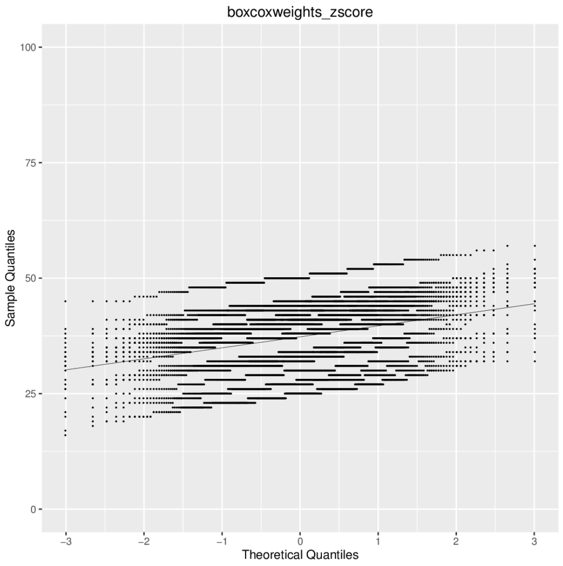

(c) Total quality score: 2

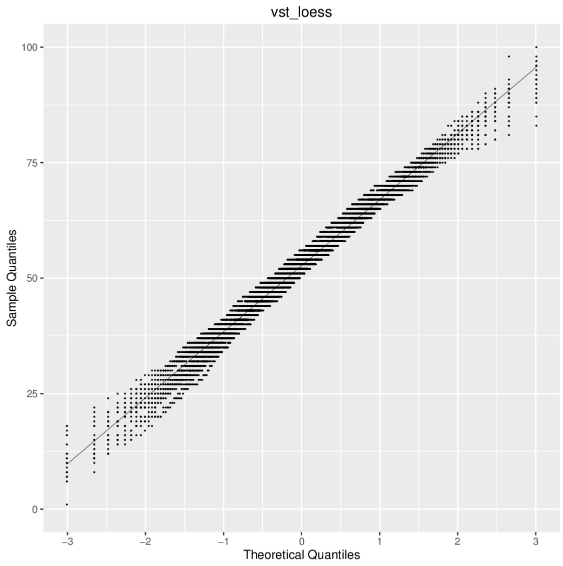

(d) Total quality score: 5

Figure S1: QQ-plots of test statistics of the Mann-Whitney U test, drawn subsamples of the two case groups, raw data, 25 replications. Used method (top) is displayed as *transformation_normalization*.

Part 2: QQ-plots, all 37 pre-processing combinations

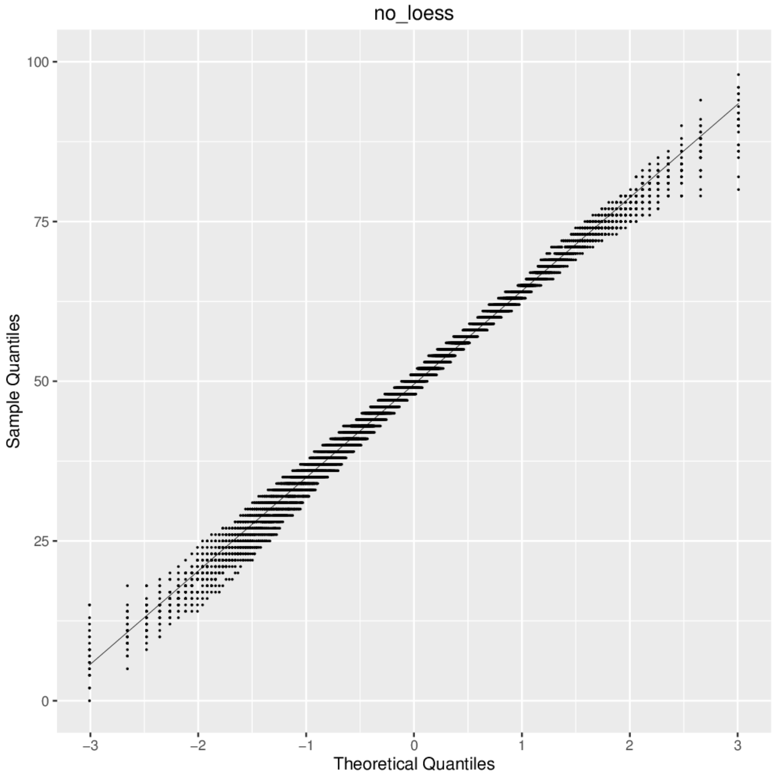

Figure S2: QQ-plot of test statistics of the Mann-Whitney U test, drawn subsamples of the two case groups, raw data, 25 replications. Used method (top) is displayed as *transformation_normalization*.

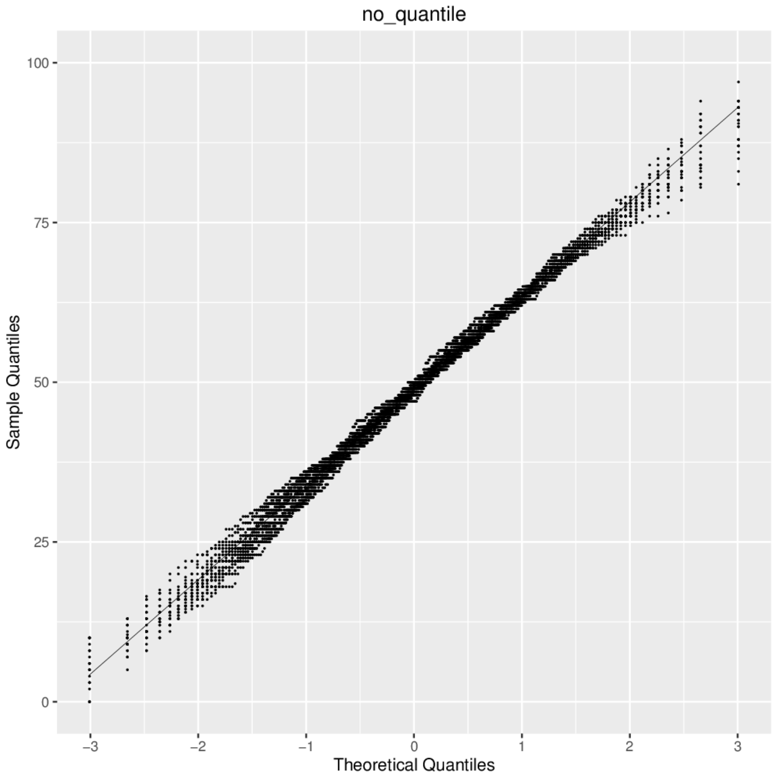

Figure S3: QQ-plot of test statistics of the Mann-Whitney U test, drawn subsamples of the two case groups, raw data, 25 replications. Used method (top) is displayed as *transformation_normalization*.

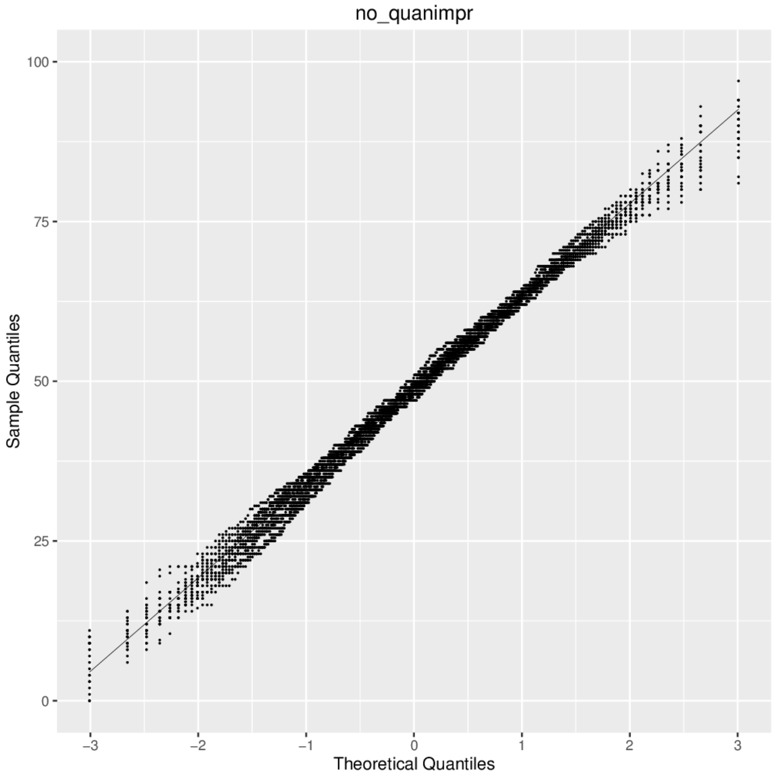

Figure S4: QQ-plot of test statistics of the Mann-Whitney U test, drawn subsamples of the two case groups, raw data, 25 replications. Used method (top) is displayed as *transformation_normalization*.

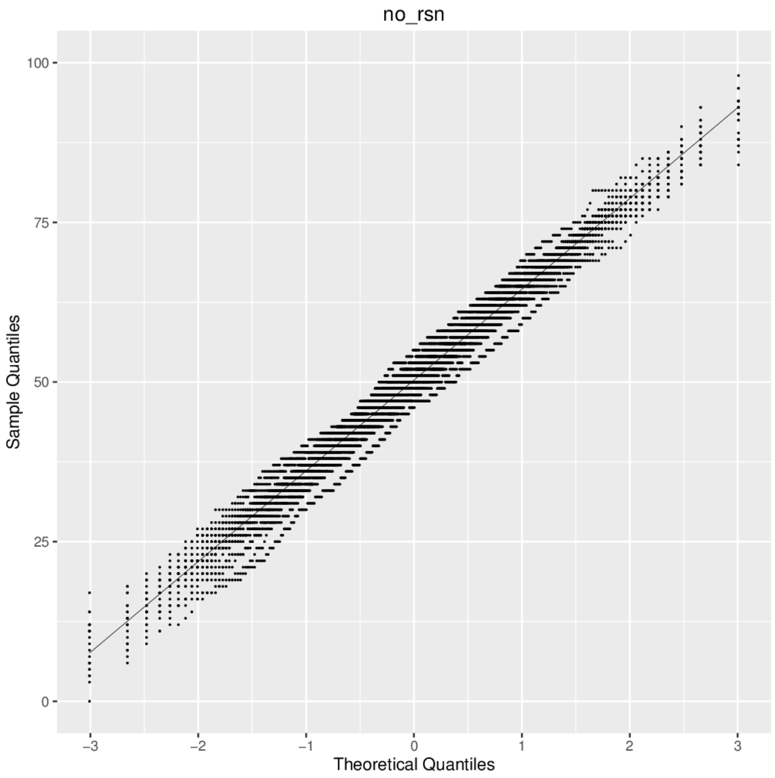

Figure S5: QQ-plot of test statistics of the Mann-Whitney U test, drawn subsamples of the two case groups, raw data, 25 replications. Used method (top) is displayed as *transformation_normalization*.

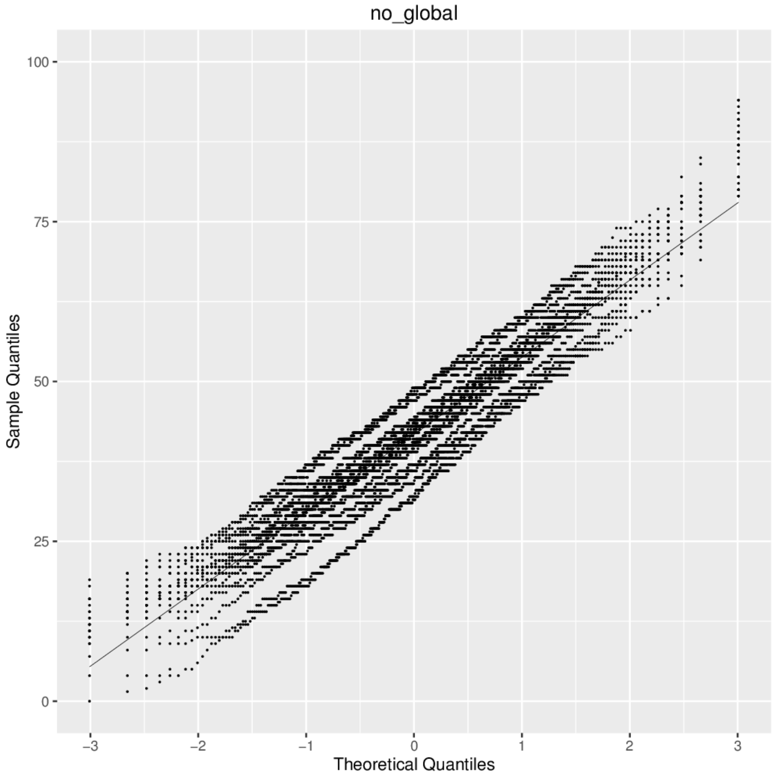

Figure S6: QQ-plot of test statistics of the Mann-Whitney U test, drawn subsamples of the two case groups, raw data, 25 replications. Used method (top) is displayed as *transformation_normalization*.

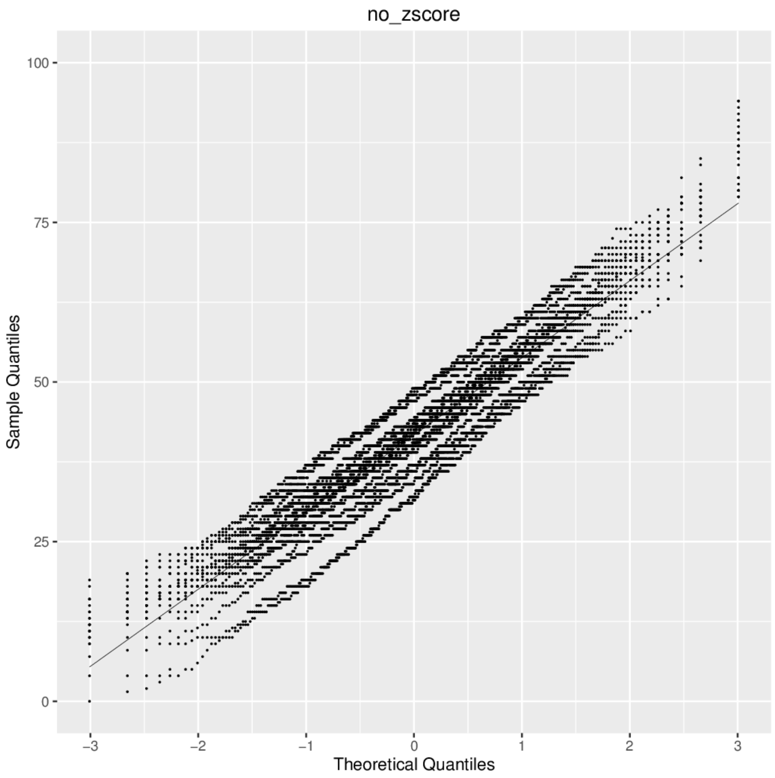

Figure S7: QQ-plot of test statistics of the Mann-Whitney U test, drawn subsamples of the two case groups, raw data, 25 replications. Used method (top) is displayed as *transformation_normalization*.

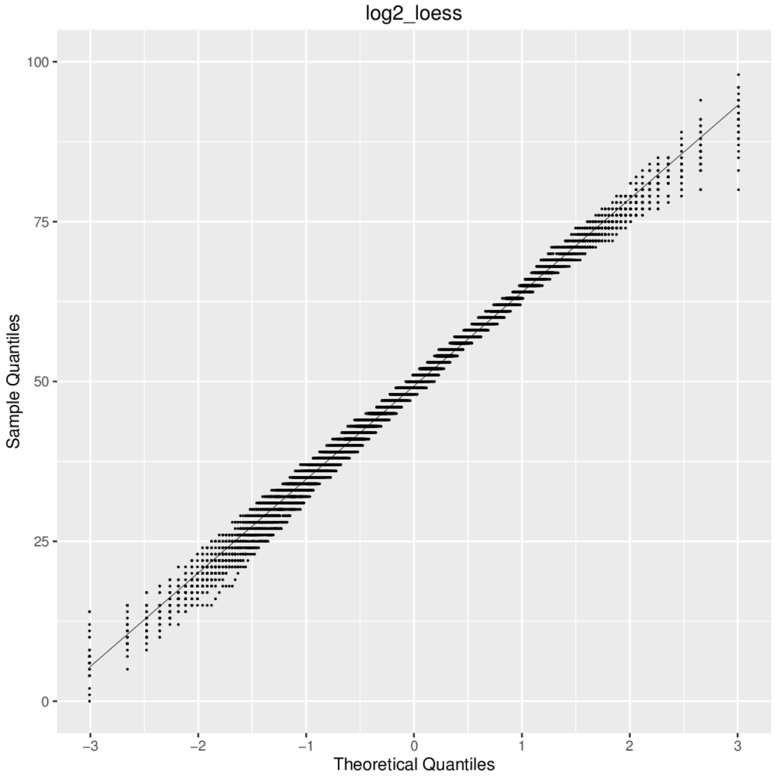

Figure S8: QQ-plot of test statistics of the Mann-Whitney U test, drawn subsamples of the two case groups, raw data, 25 replications. Used method (top) is displayed as *transformation_normalization*.

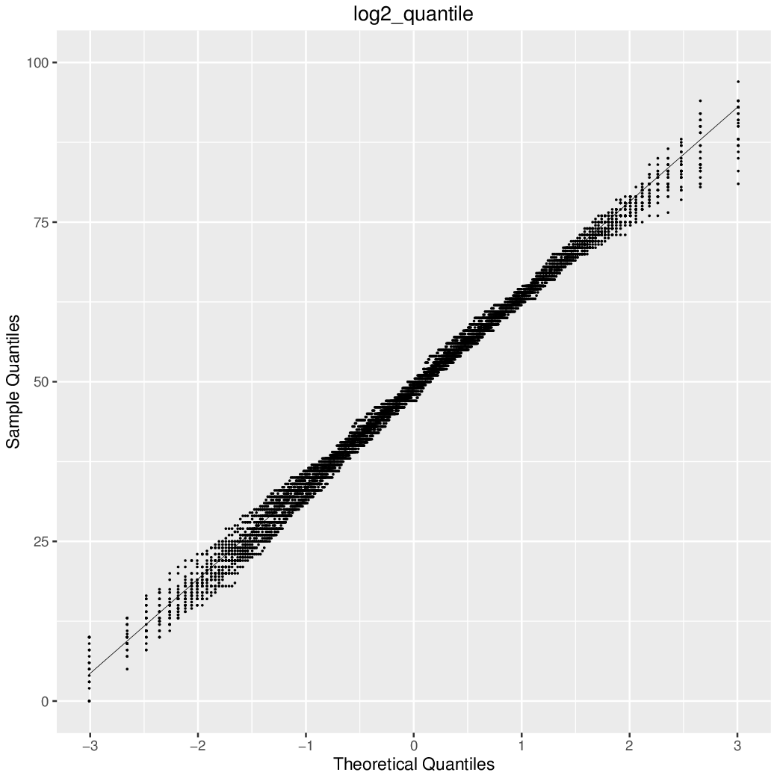

Figure S9: QQ-plot of test statistics of the Mann-Whitney U test, drawn subsamples of the two case groups, raw data, 25 replications. Used method (top) is displayed as *transformation_normalization*.

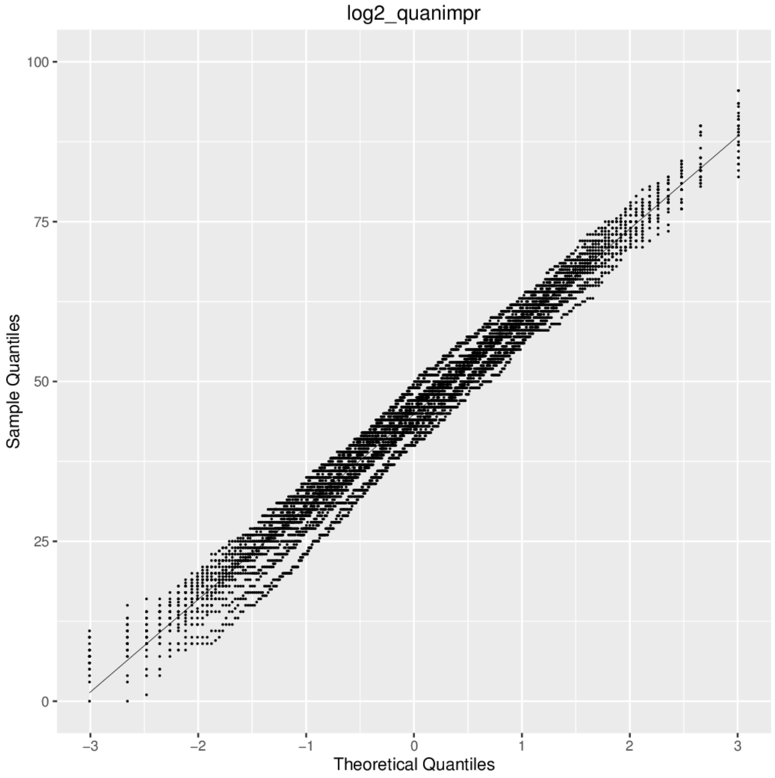

Figure S10: QQ-plot of test statistics of the Mann-Whitney U test, drawn subsamples of the two case groups, raw data, 25 replications. Used method (top) is displayed as *transformation_normalization*.

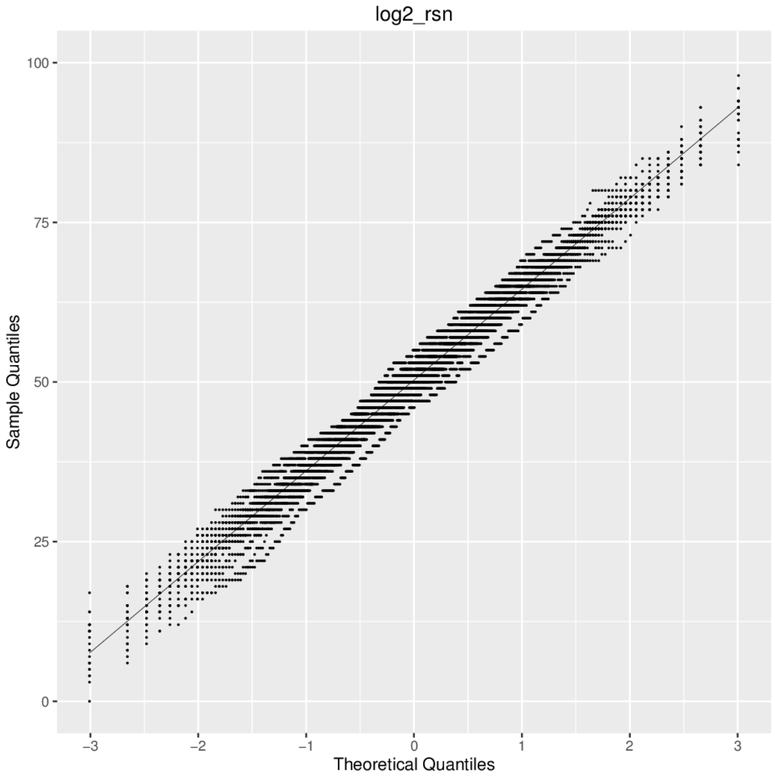

Figure S11: QQ-plot of test statistics of the Mann-Whitney U test, drawn subsamples of the two case groups, raw data, 25 replications. Used method (top) is displayed as *transformation_normalization*.

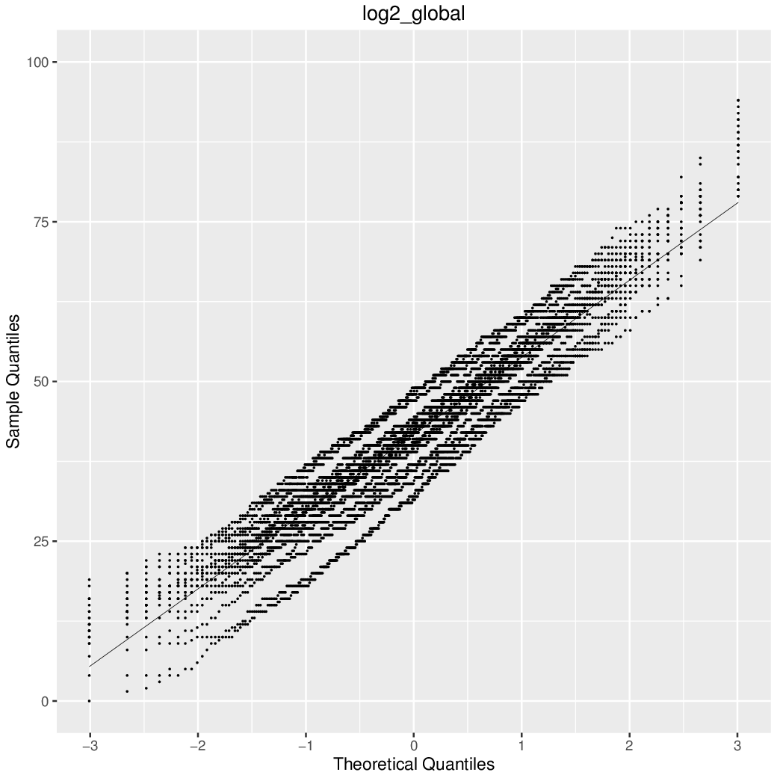

Figure S12: QQ-plot of test statistics of the Mann-Whitney U test, drawn subsamples of the two case groups, raw data, 25 replications. Used method (top) is displayed as *transformation_normalization*.

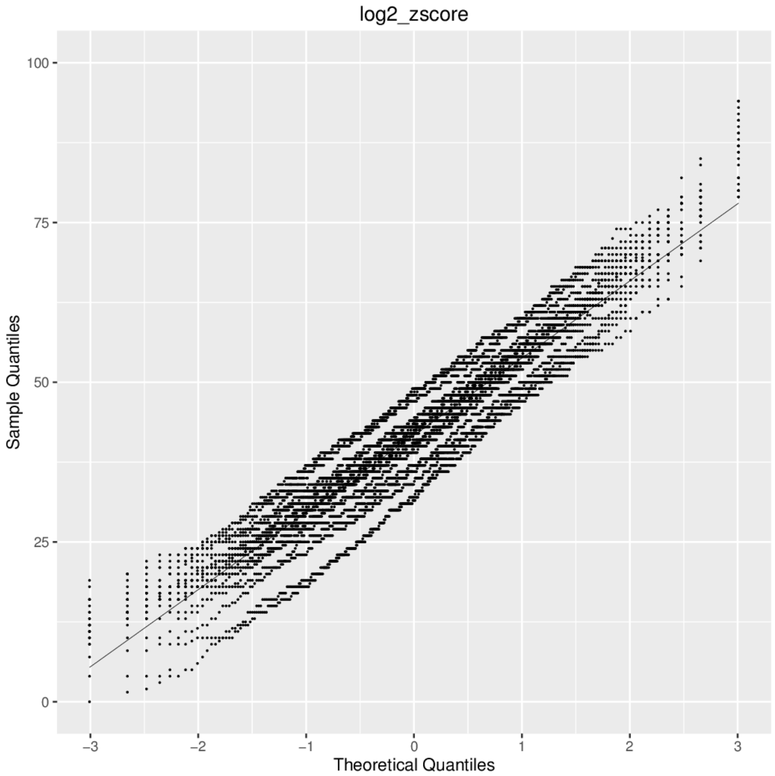

Figure S13: QQ-plot of test statistics of the Mann-Whitney U test, drawn subsamples of the two case groups, raw data, 25 replications. Used method (top) is displayed as *transformation_normalization*.

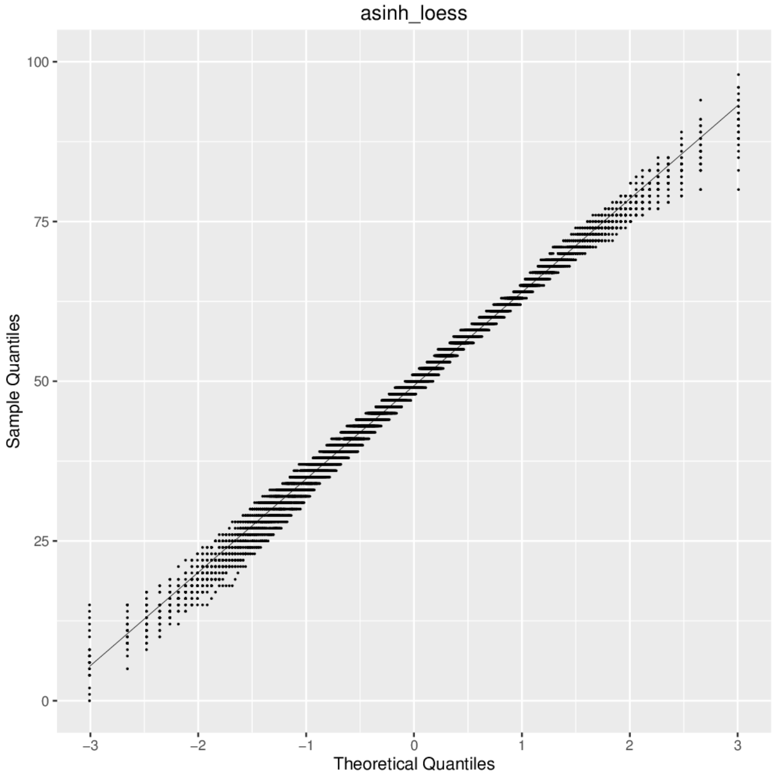

Figure S14: QQ-plot of test statistics of the Mann-Whitney U test, drawn subsamples of the two case groups, raw data, 25 replications. Used method (top) is displayed as *transformation_normalization*.

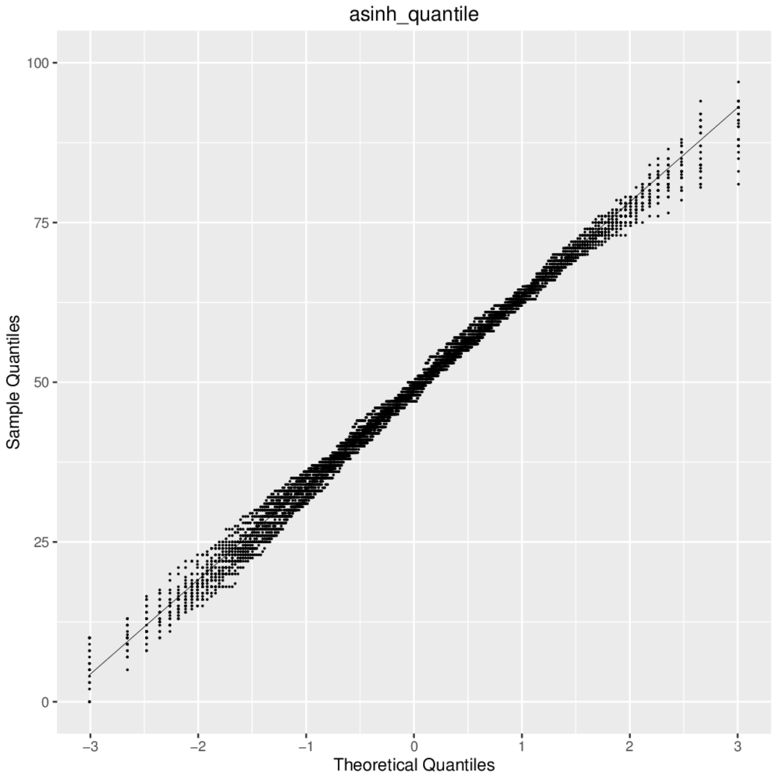

Figure S15: QQ-plot of test statistics of the Mann-Whitney U test, drawn subsamples of the two case groups, raw data, 25 replications. Used method (top) is displayed as *transformation_normalization*.

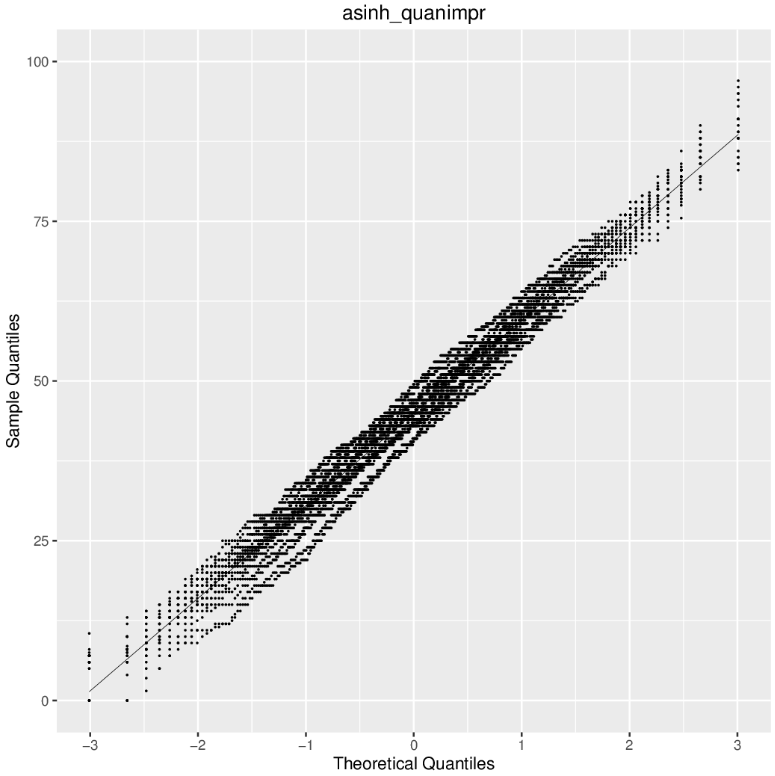

Figure S16: QQ-plot of test statistics of the Mann-Whitney U test, drawn subsamples of the two case groups, raw data, 25 replications. Used method (top) is displayed as *transformation_normalization*.

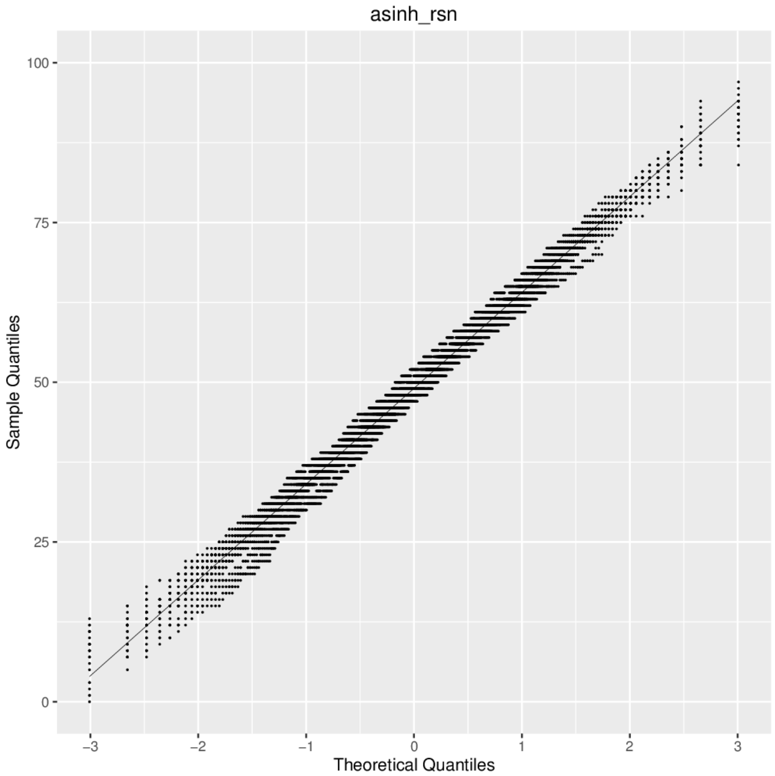

Figure S17: QQ-plot of test statistics of the Mann-Whitney U test, drawn subsamples of the two case groups, raw data, 25 replications. Used method (top) is displayed as *transformation_normalization*.

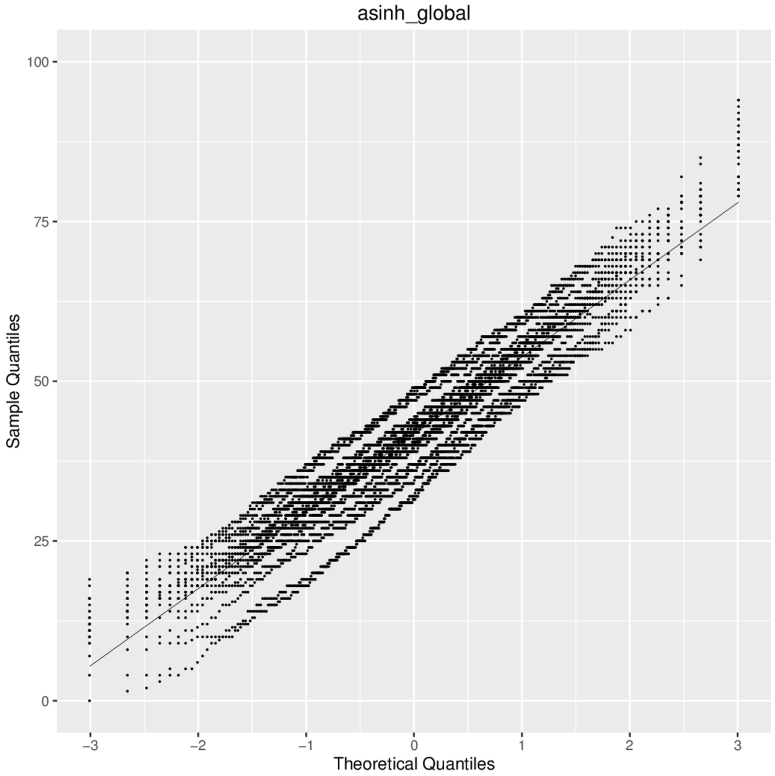

Figure S18: QQ-plot of test statistics of the Mann-Whitney U test, drawn subsamples of the two case groups, raw data, 25 replications. Used method (top) is displayed as *transformation_normalization*.

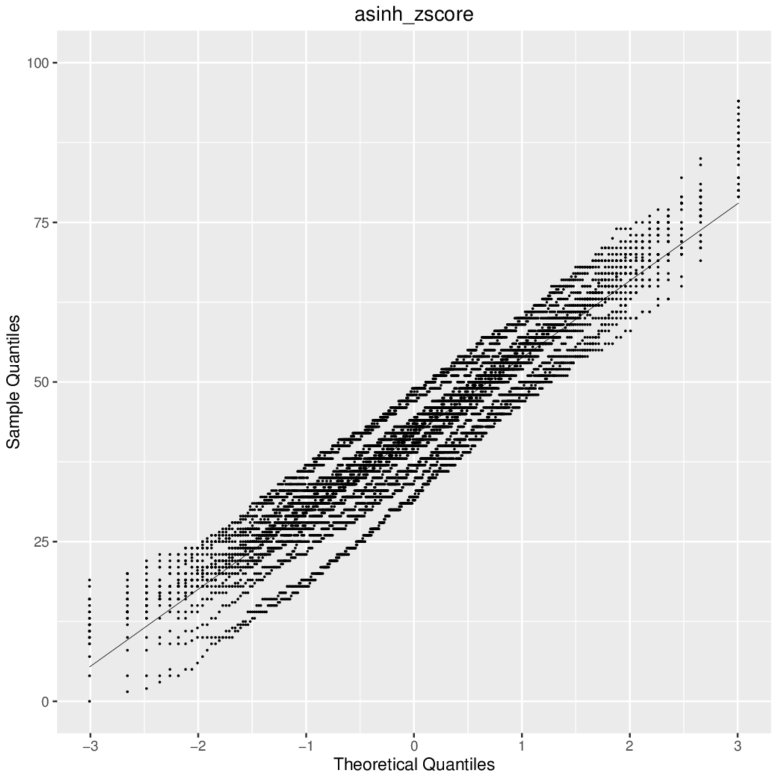

Figure S19: QQ-plot of test statistics of the Mann-Whitney U test, drawn subsamples of the two case groups, raw data, 25 replications. Used method (top) is displayed as *transformation_normalization*.

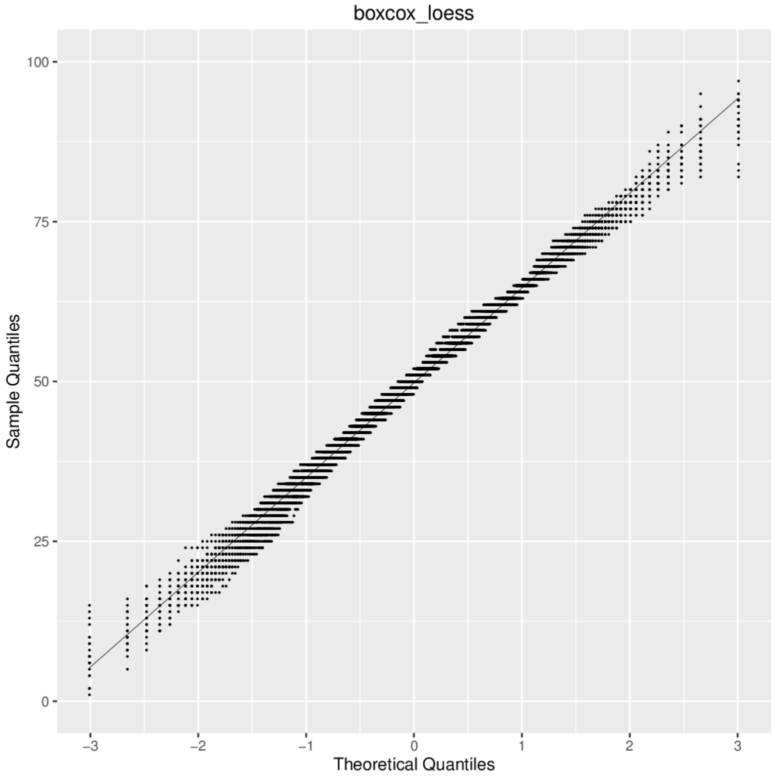

Figure S20: QQ-plot of test statistics of the Mann-Whitney U test, drawn subsamples of the two case groups, raw data, 25 replications. Used method (top) is displayed as *transformation_normalization*.

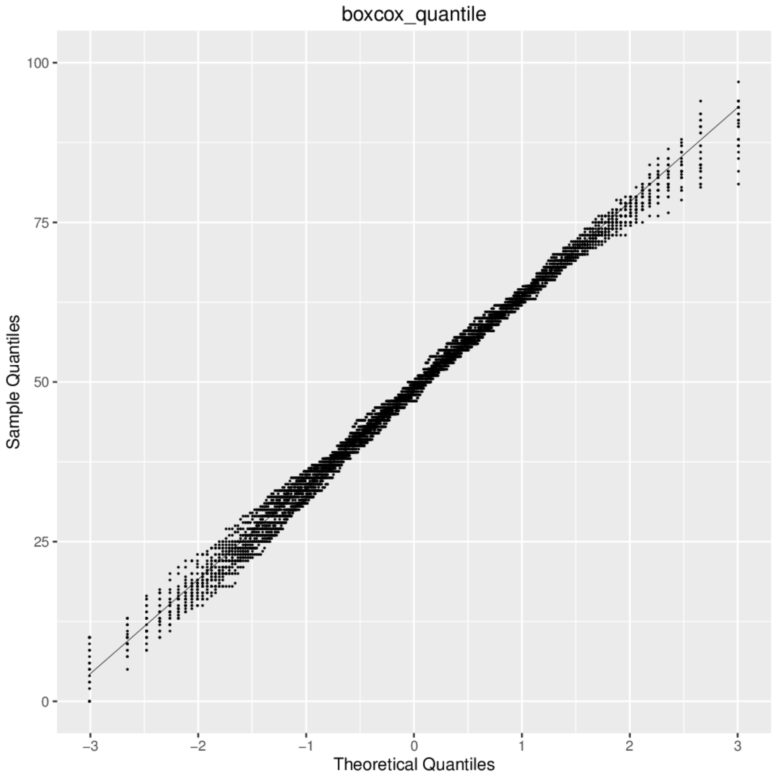

Figure S21: QQ-plot of test statistics of the Mann-Whitney U test, drawn subsamples of the two case groups, raw data, 25 replications. Used method (top) is displayed as *transformation_normalization*.

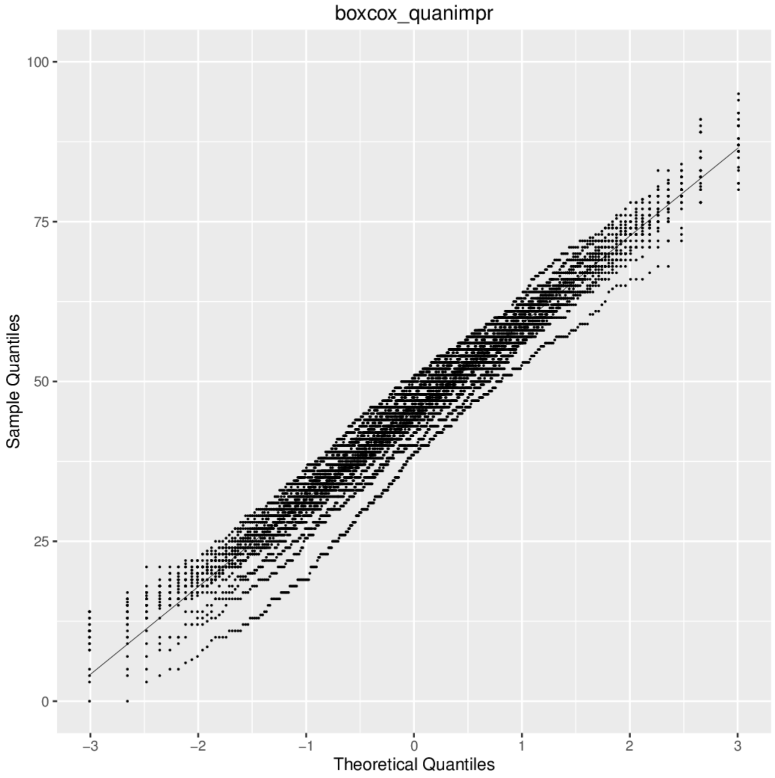

Figure S22: QQ-plot of test statistics of the Mann-Whitney U test, drawn subsamples of the two case groups, raw data, 25 replications. Used method (top) is displayed as *transformation_normalization*.

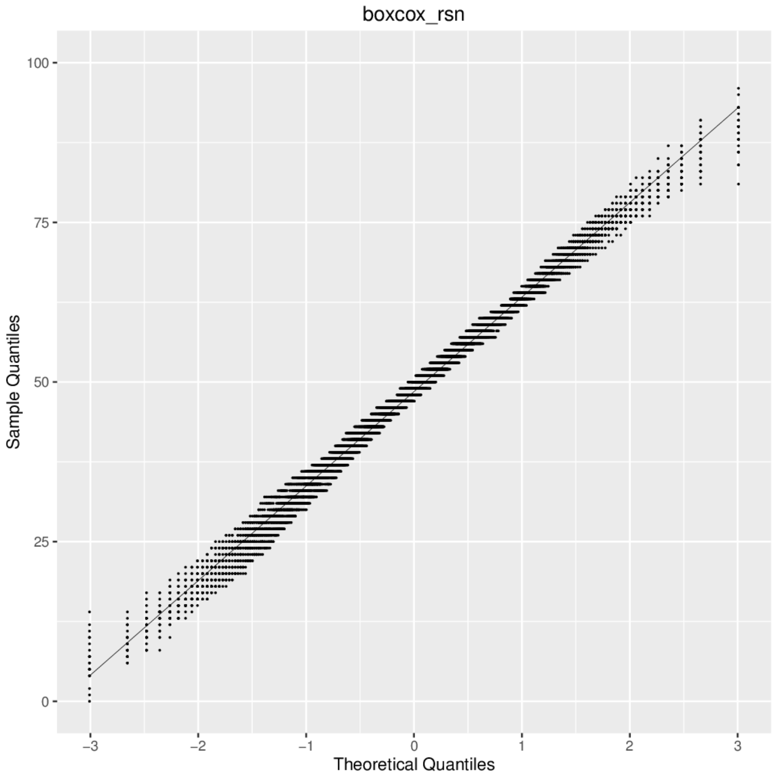

Figure S23: QQ-plot of test statistics of the Mann-Whitney U test, drawn subsamples of the two case groups, raw data, 25 replications. Used method (top) is displayed as *transformation_normalization*.

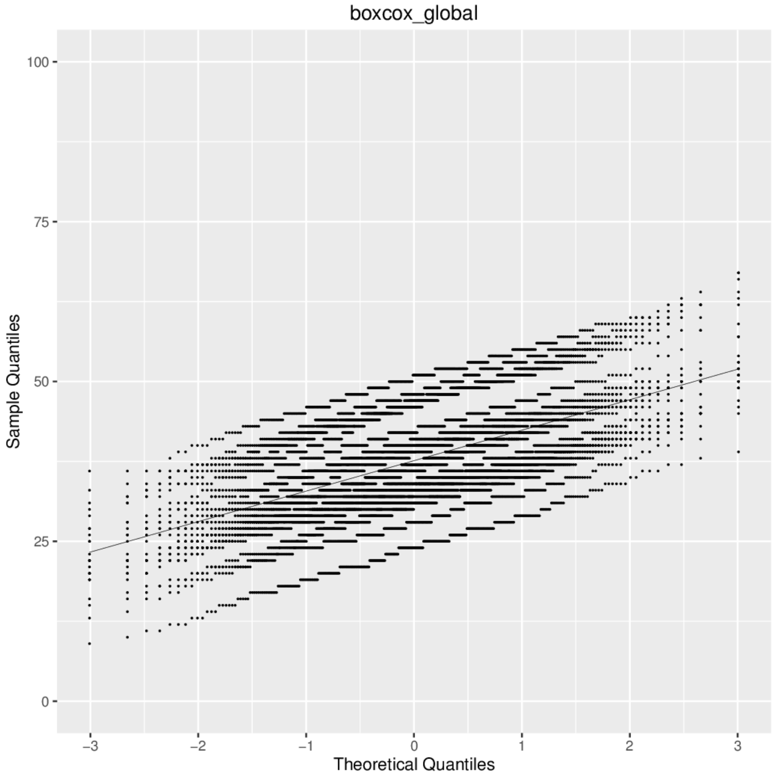

Figure S24: QQ-plot of test statistics of the Mann-Whitney U test, drawn subsamples of the two case groups, raw data, 25 replications. Used method (top) is displayed as *transformation_normalization*.

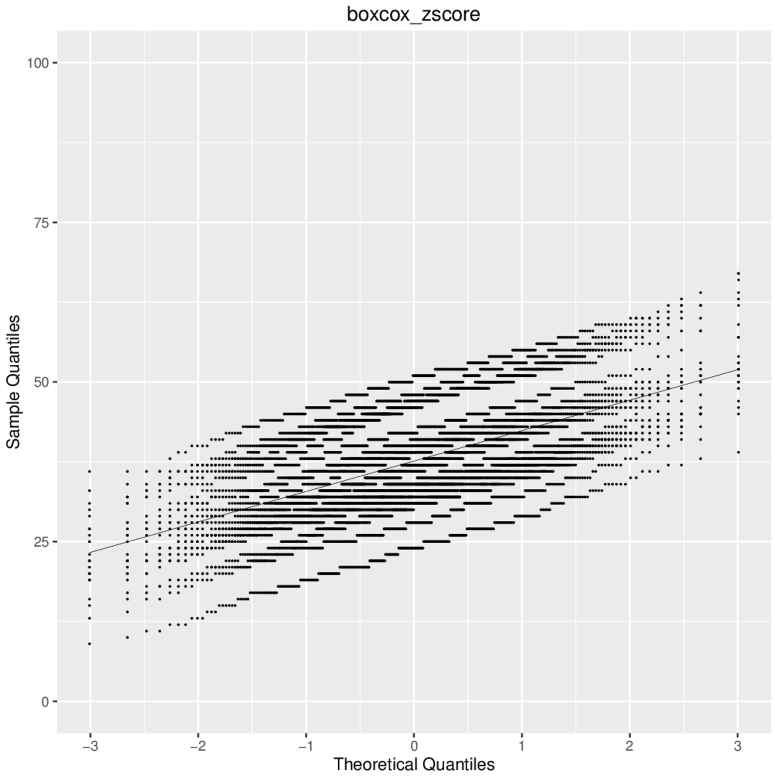

Figure S25: QQ-plot of test statistics of the Mann-Whitney U test, drawn subsamples of the two case groups, raw data, 25 replications. Used method (top) is displayed as *transformation_normalization*.

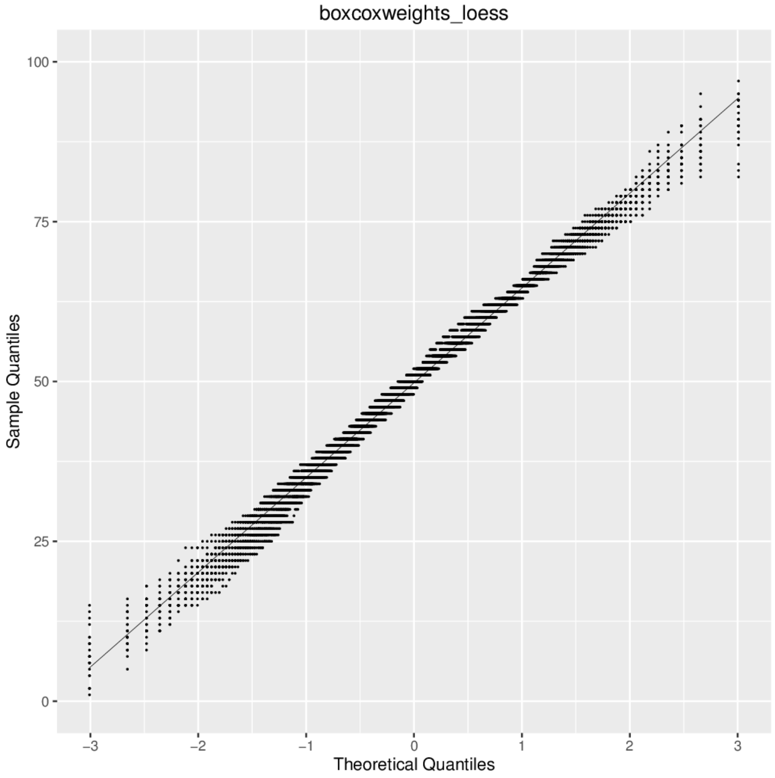

Figure S26: QQ-plot of test statistics of the Mann-Whitney U test, drawn subsamples of the two case groups, raw data, 25 replications. Used method (top) is displayed as *transformation_normalization*.

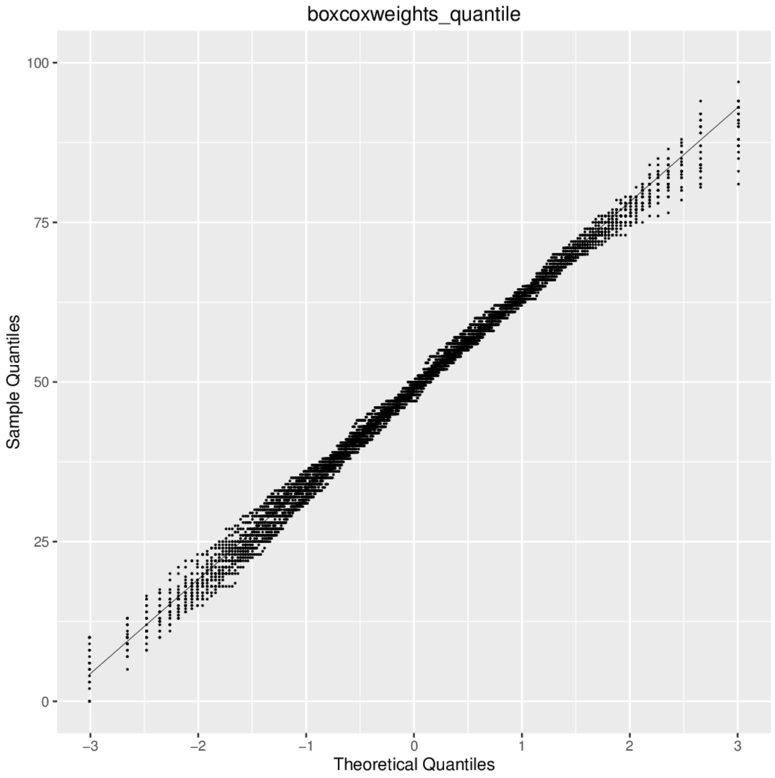

Figure S27: QQ-plot of test statistics of the Mann-Whitney U test, drawn subsamples of the two case groups, raw data, 25 replications. Used method (top) is displayed as *transformation_normalization*.

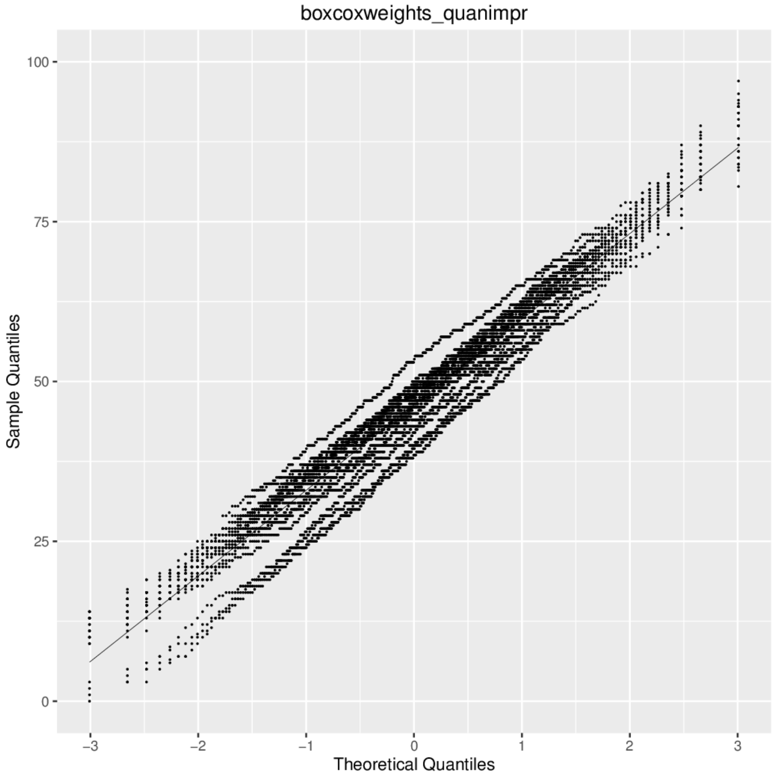

Figure S28: QQ-plot of test statistics of the Mann-Whitney U test, drawn subsamples of the two case groups, raw data, 25 replications. Used method (top) is displayed as *transformation_normalization*.

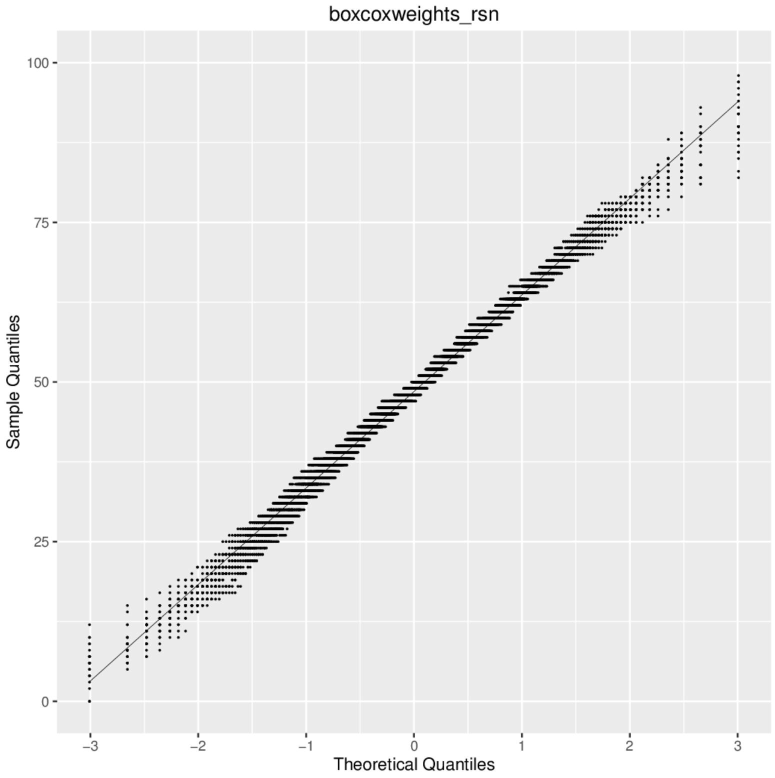

Figure S29: QQ-plot of test statistics of the Mann-Whitney U test, drawn subsamples of the two case groups, raw data, 25 replications. Used method (top) is displayed as *transformation_normalization*.

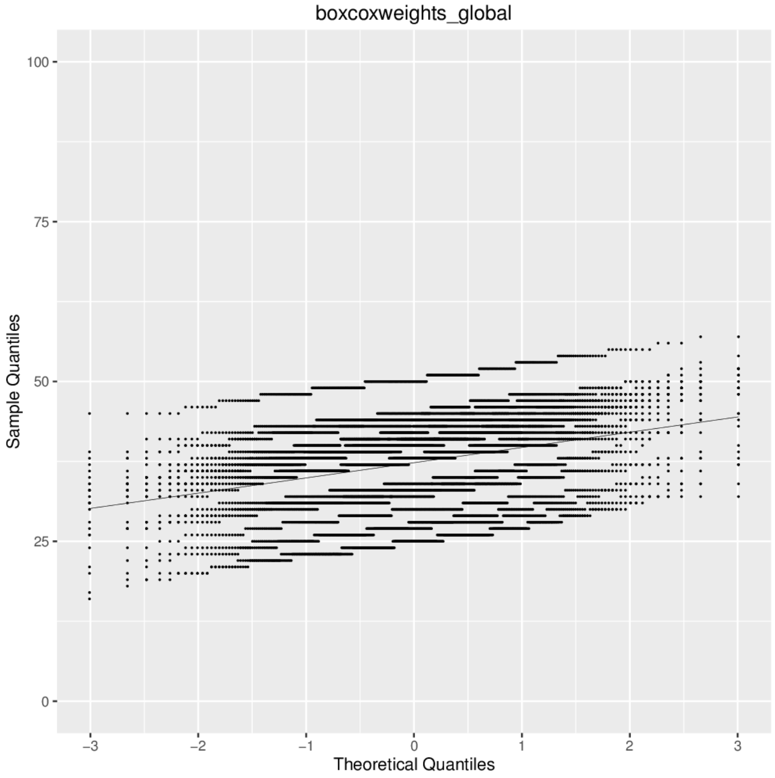

Figure S30: QQ-plot of test statistics of the Mann-Whitney U test, drawn subsamples of the two case groups, raw data, 25 replications. Used method (top) is displayed as *transformation_normalization*.

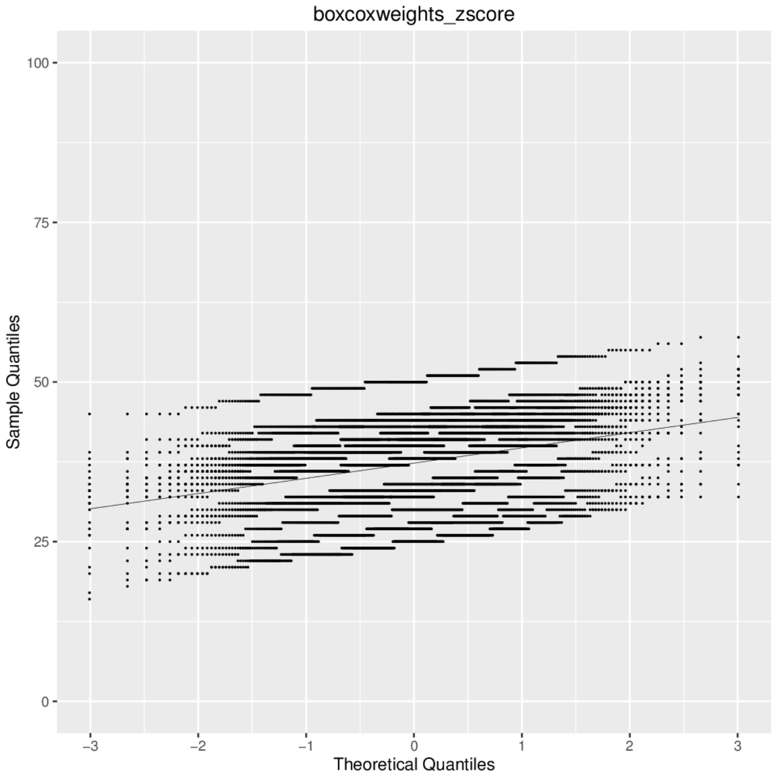

Figure S31: QQ-plot of test statistics of the Mann-Whitney U test, drawn subsamples of the two case groups, raw data, 25 replications. Used method (top) is displayed as *transformation_normalization*.

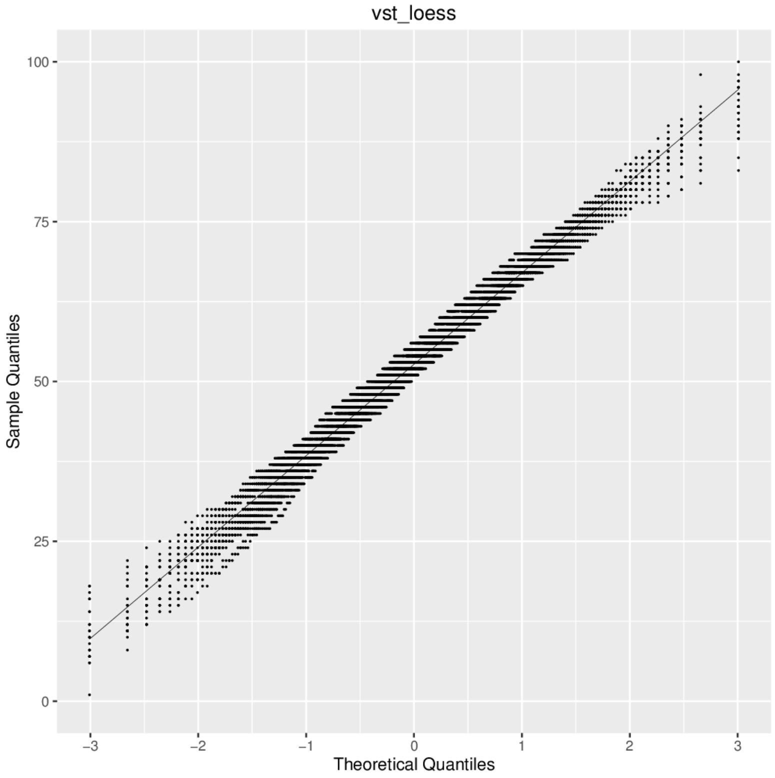

Figure S32: QQ-plot of test statistics of the Mann-Whitney U test, drawn subsamples of the two case groups, raw data, 25 replications. Used method (top) is displayed as *transformation_normalization*.

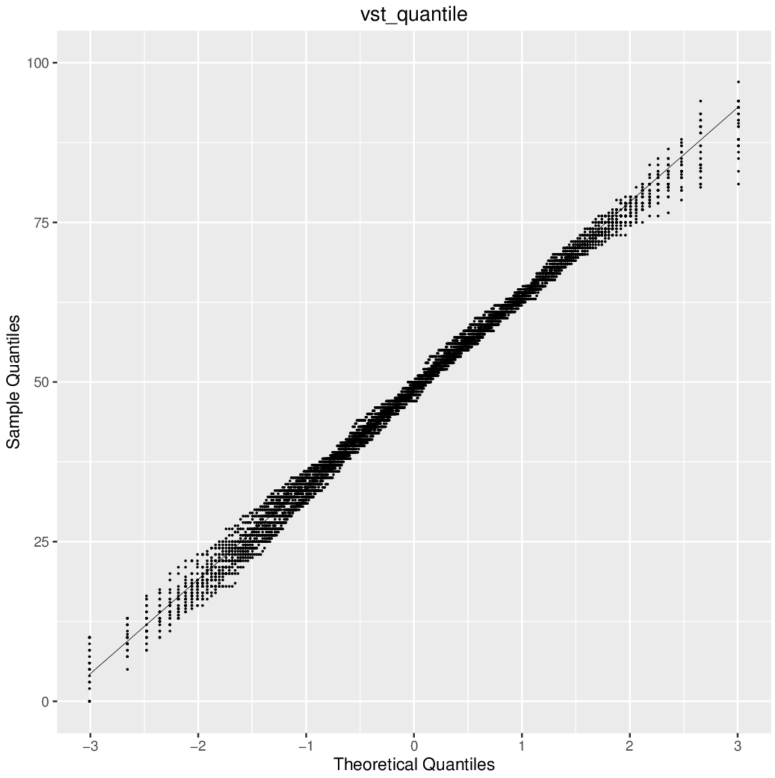

Figure S33: QQ-plot of test statistics of the Mann-Whitney U test, drawn subsamples of the two case groups, raw data, 25 replications. Used method (top) is displayed as *transformation_normalization*.

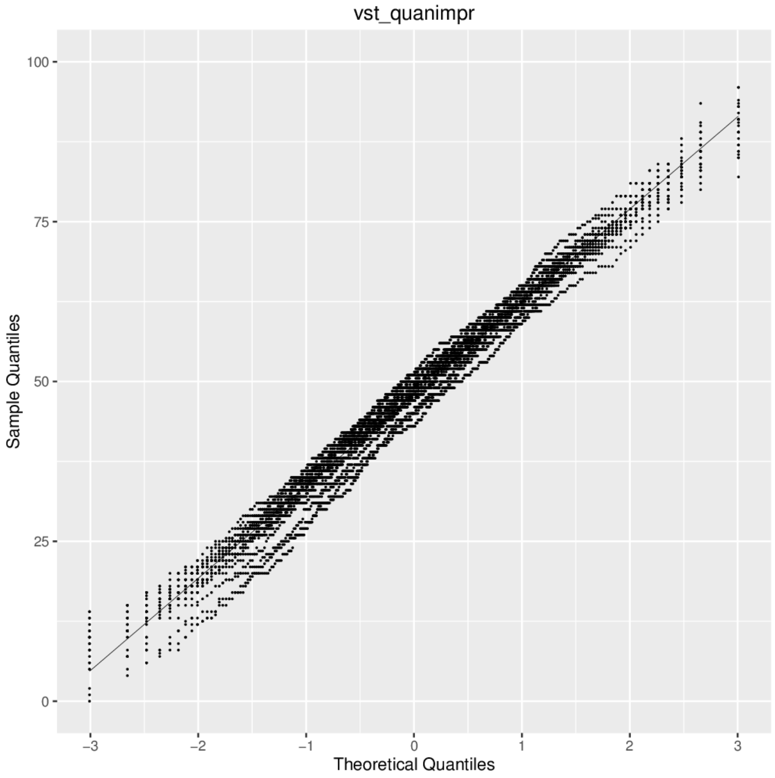

Figure S34: QQ-plot of test statistics of the Mann-Whitney U test, drawn subsamples of the two case groups, raw data, 25 replications. Used method (top) is displayed as *transformation_normalization*.

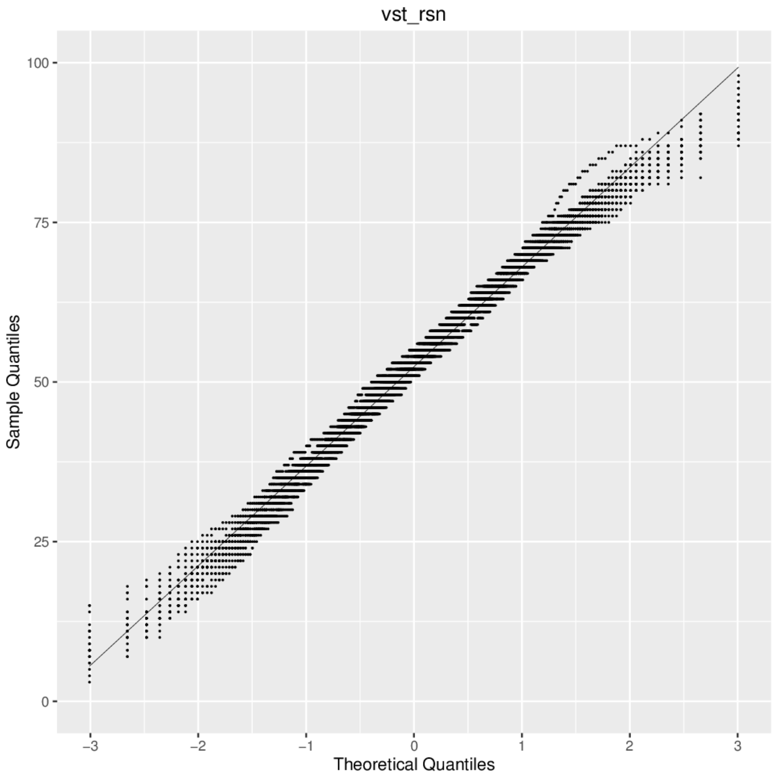

Figure S35: QQ-plot of test statistics of the Mann-Whitney U test, drawn subsamples of the two case groups, raw data, 25 replications. Used method (top) is displayed as *transformation_normalization*.

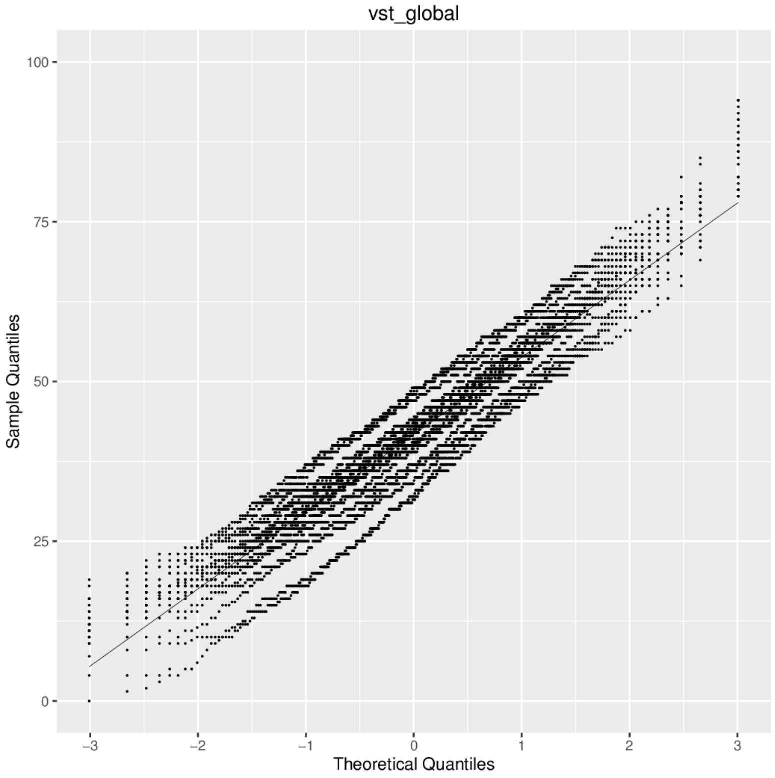

Figure S36: QQ-plot of test statistics of the Mann-Whitney U test, drawn subsamples of the two case groups, raw data, 25 replications. Used method (top) is displayed as *transformation_normalization*.

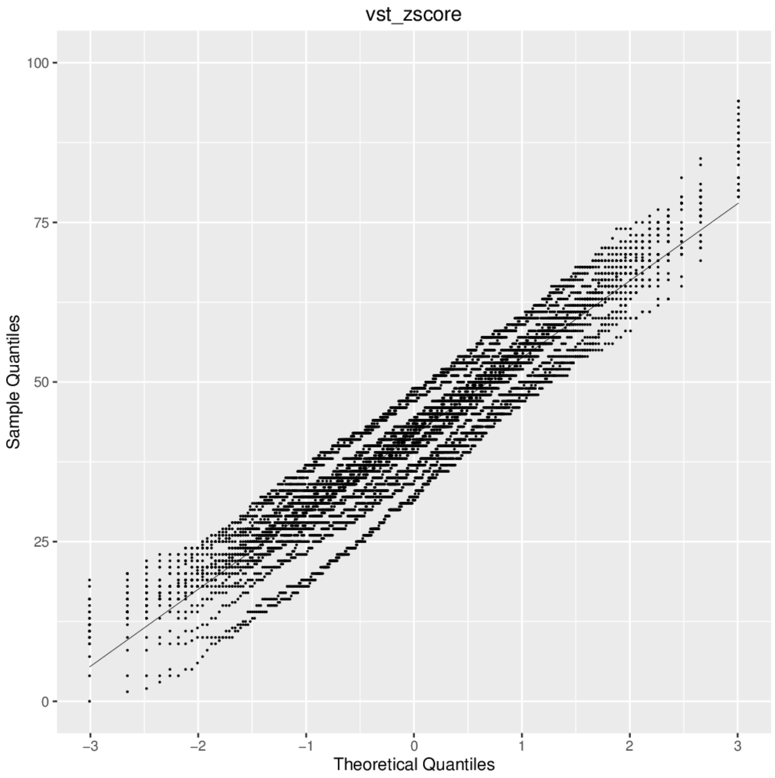

Figure S37: QQ-plot of test statistics of the Mann-Whitney U test, drawn subsamples of the two case groups, raw data, 25 replications. Used method (top) is displayed as *transformation_normalization*.

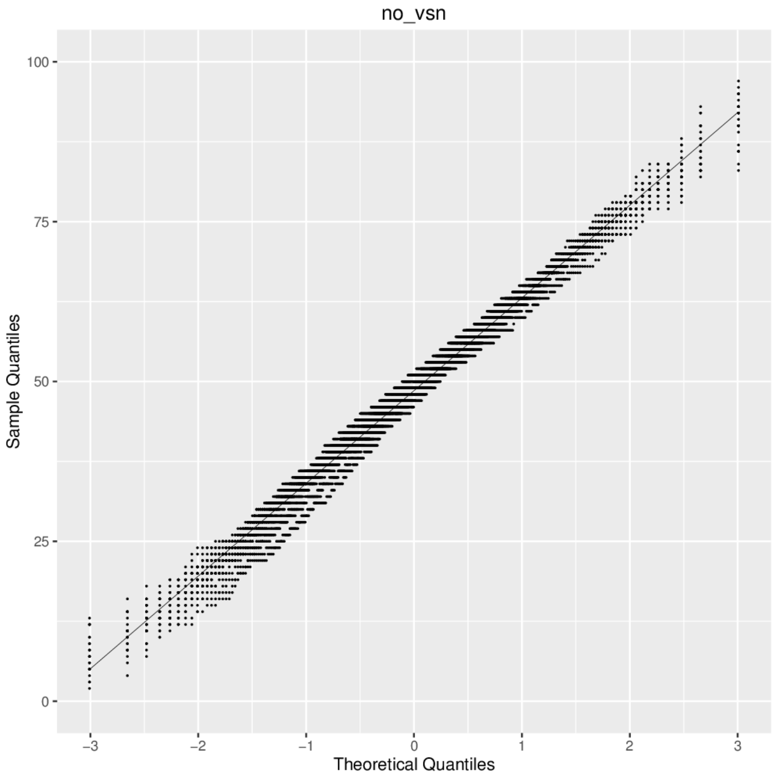

Figure S38: QQ-plot of test statistics of the Mann-Whitney U test, drawn subsamples of the two case groups, raw data, 25 replications. Used method (top) is displayed as *transformation_normalization*.

Part 3: Plots of all 37 pre-processing methods investigated in this work and shown to blinded readers

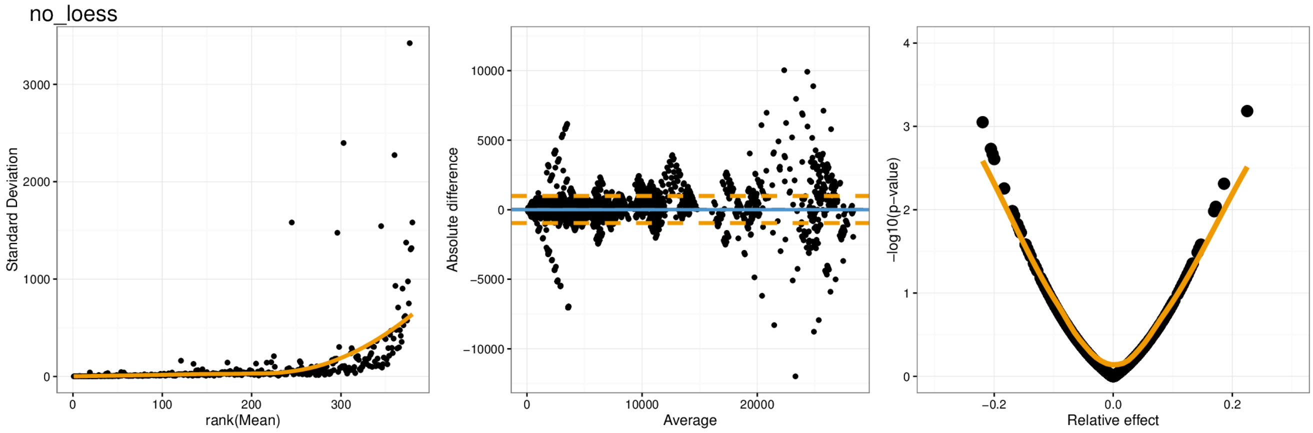

Figure S39: Mean-SD plot (left), Bland-Altman plot (middle), Volcano plot (right). Used method (top left) is displayed as *transformation_normalization*.

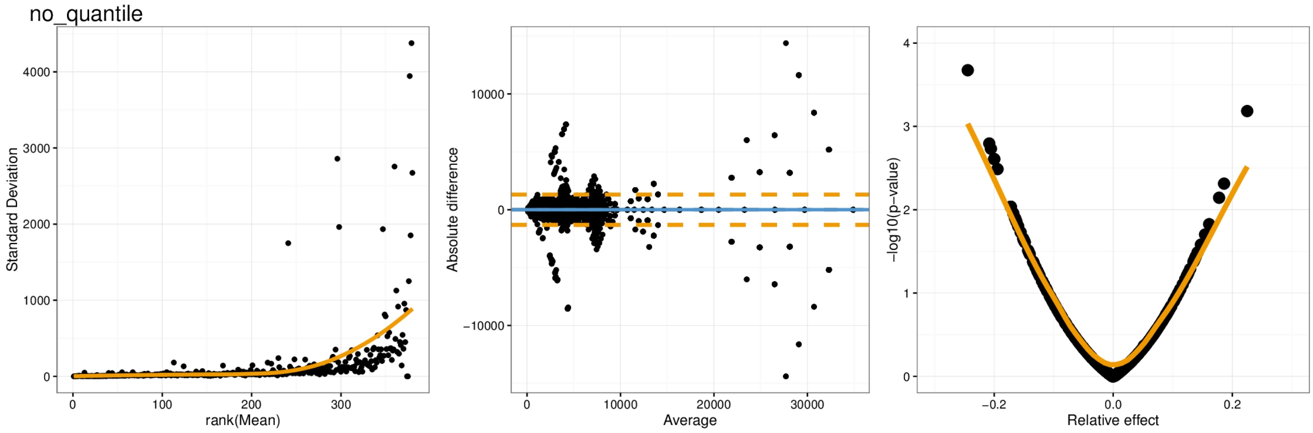

Figure S40: Mean-SD plot (left), Bland-Altman plot (middle), Volcano plot (right). Used method (top left) is displayed as *transformation_normalization*.

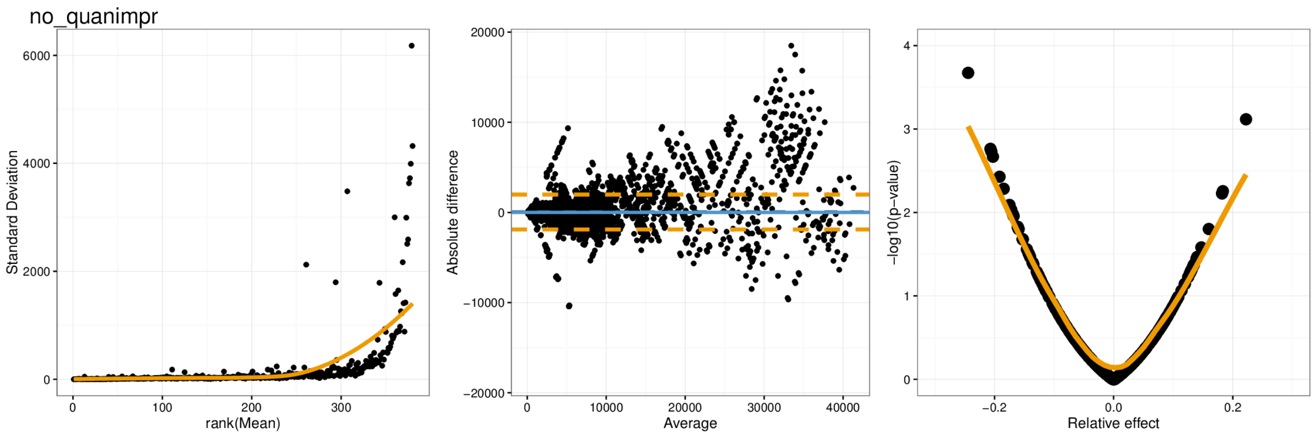

Figure S41: Mean-SD plot (left), Bland-Altman plot (middle), Volcano plot (right). Used method (top left) is displayed as *transformation_normalization*.

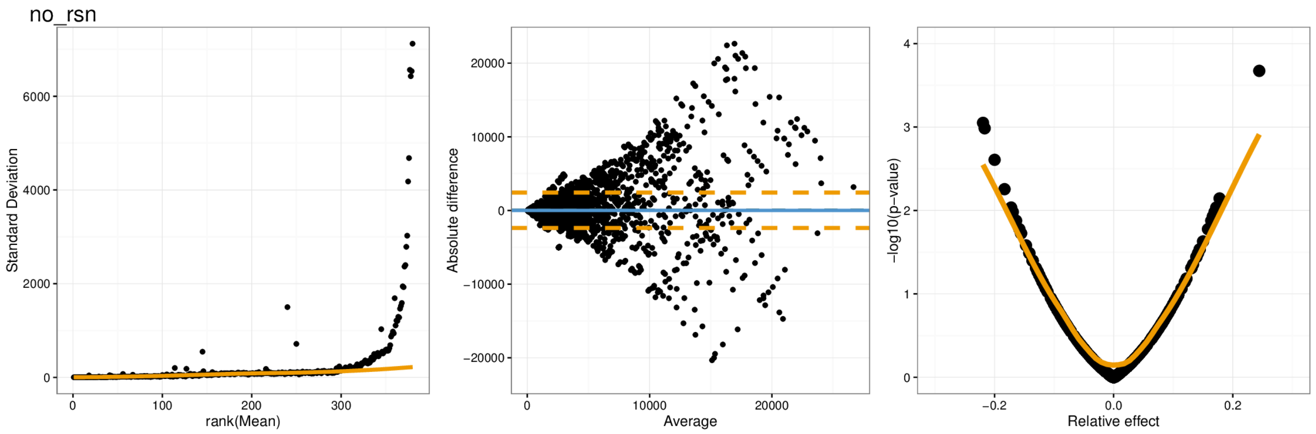

Figure S42: Mean-SD plot (left), Bland-Altman plot (middle), Volcano plot (right). Used method (top left) is displayed as *transformation_normalization*.

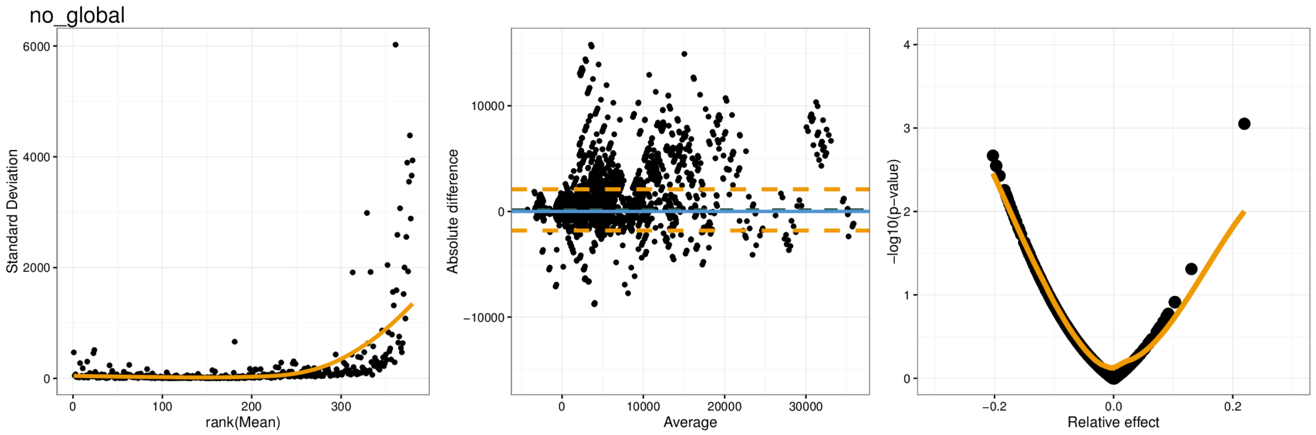

Figure S43: Mean-SD plot (left), Bland-Altman plot (middle), Volcano plot (right). Used method (top left) is displayed as *transformation_normalization*.

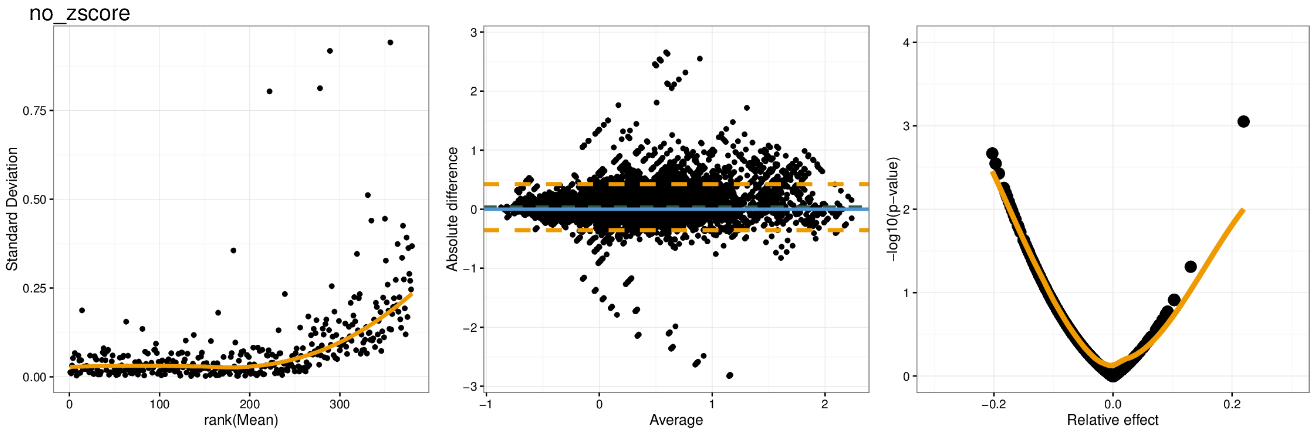

Figure S44: Mean-SD plot (left), Bland-Altman plot (middle), Volcano plot (right). Used method (top left) is displayed as *transformation_normalization*.

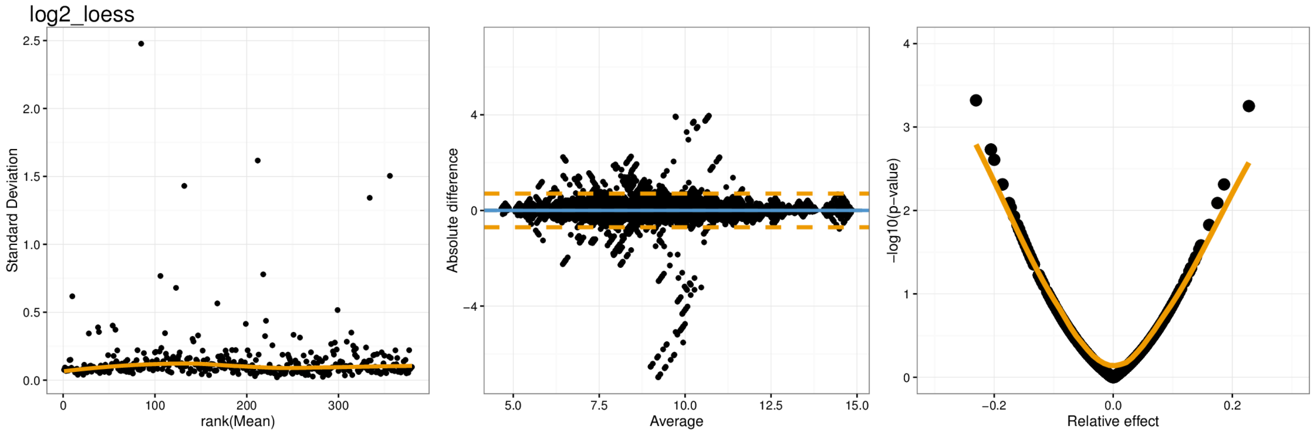

Figure S45: Mean-SD plot (left), Bland-Altman plot (middle), Volcano plot (right). Used method (top left) is displayed as *transformation_normalization*.

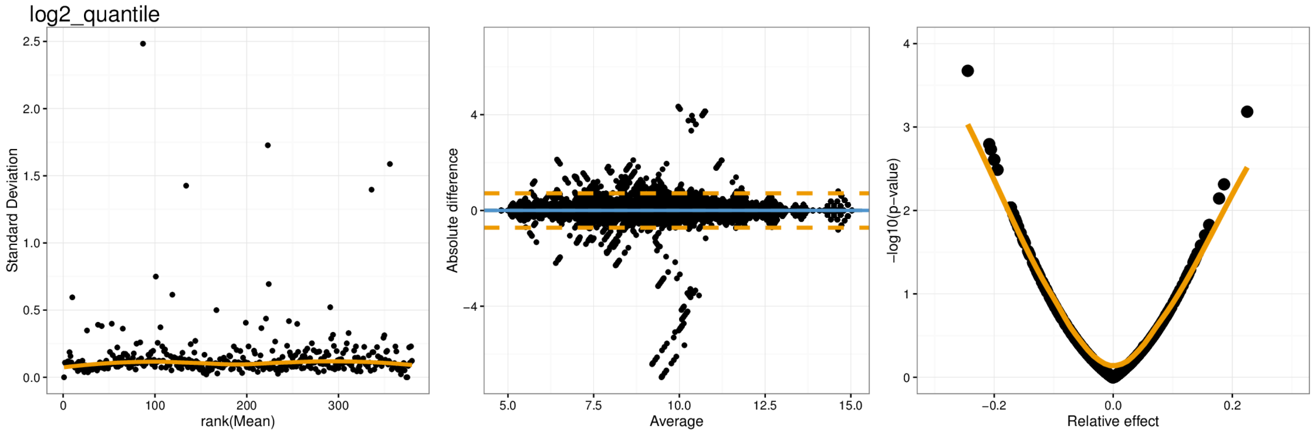

Figure S46: Mean-SD plot (left), Bland-Altman plot (middle), Volcano plot (right). Used method (top left) is displayed as *transformation_normalization*.

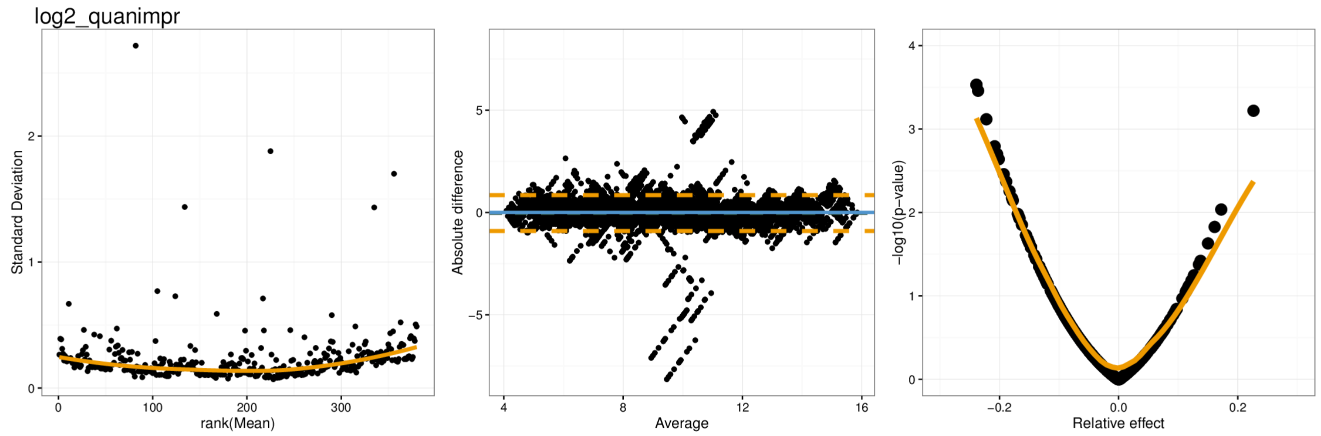

Figure S47: Mean-SD plot (left), Bland-Altman plot (middle), Volcano plot (right). Used method (top left) is displayed as *transformation_normalization*.

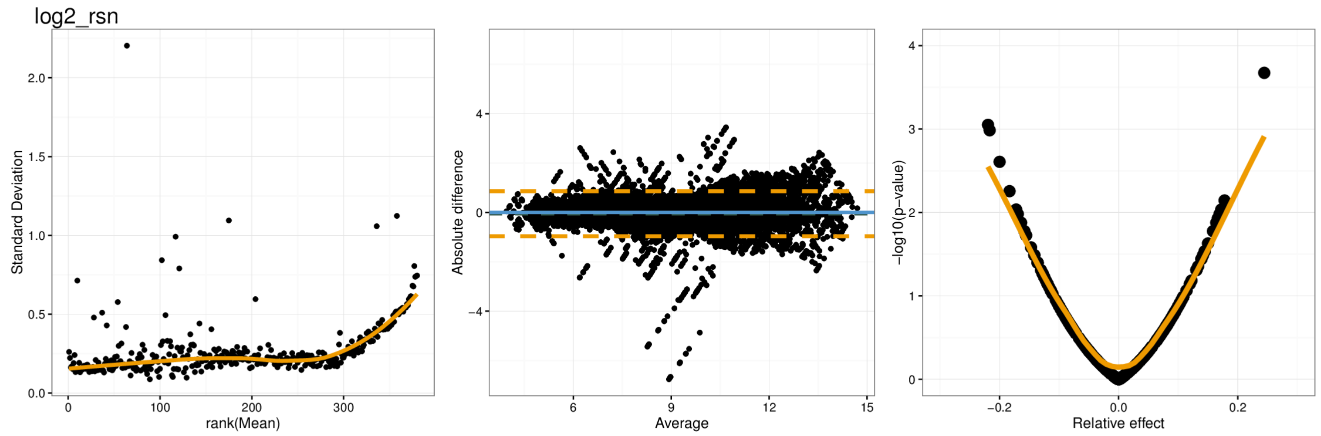

Figure S48: Mean-SD plot (left), Bland-Altman plot (middle), Volcano plot (right). Used method (top left) is displayed as *transformation_normalization*.

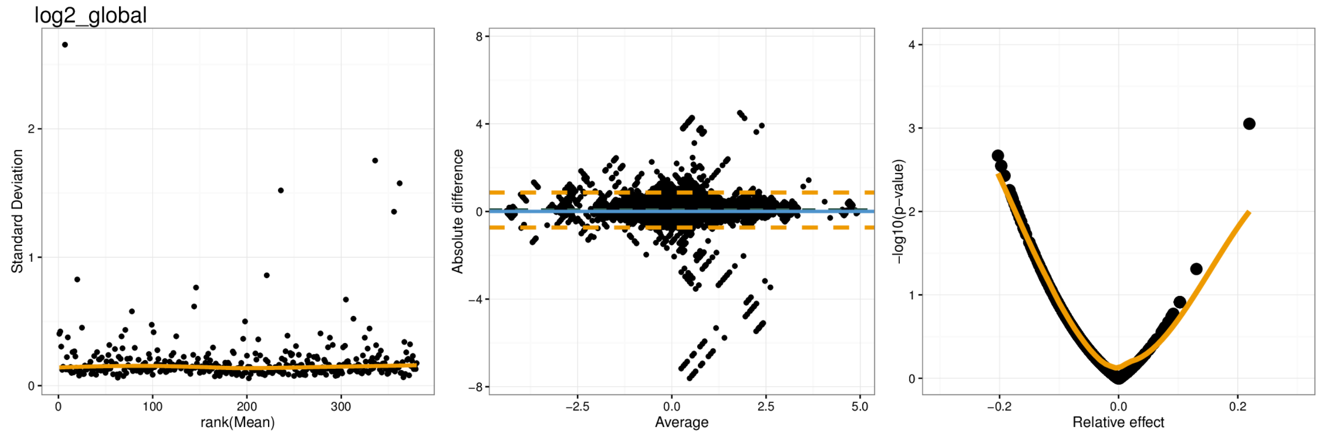

Figure S49: Mean-SD plot (left), Bland-Altman plot (middle), Volcano plot (right). Used method (top left) is displayed as *transformation_normalization*.

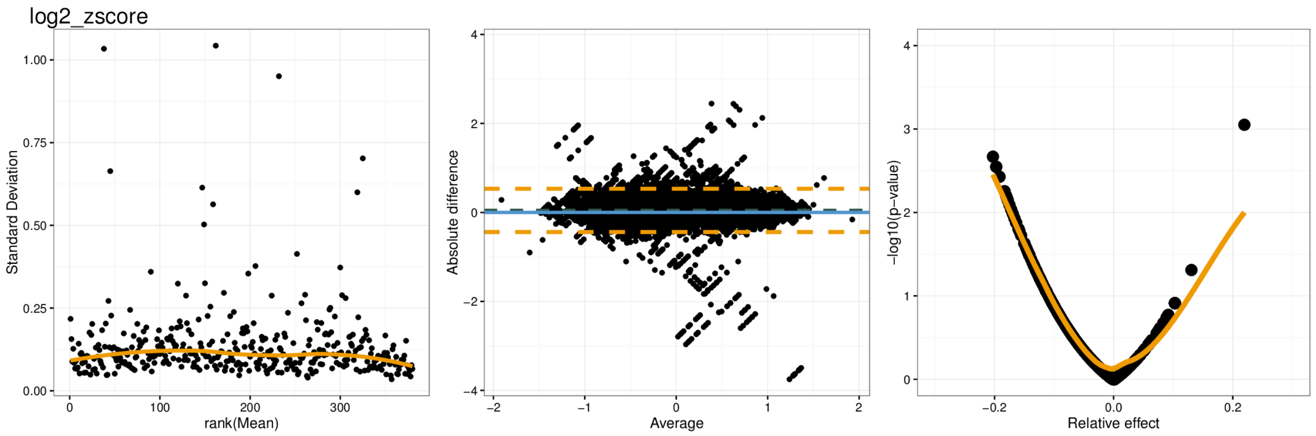

Figure S50: Mean-SD plot (left), Bland-Altman plot (middle), Volcano plot (right). Used method (top left) is displayed as *transformation_normalization*.

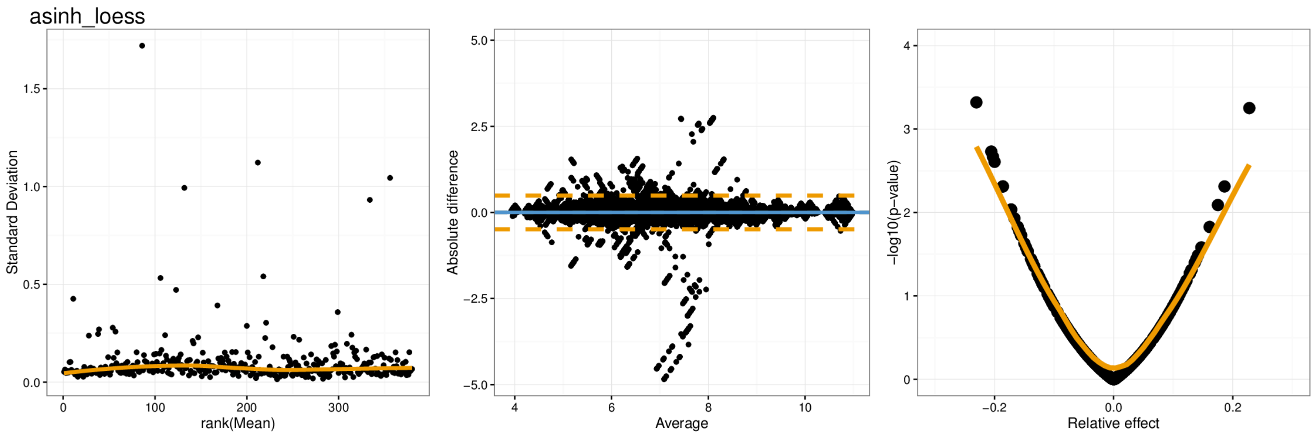

Figure S51: Mean-SD plot (left), Bland-Altman plot (middle), Volcano plot (right). Used method (top left) is displayed as *transformation_normalization*.

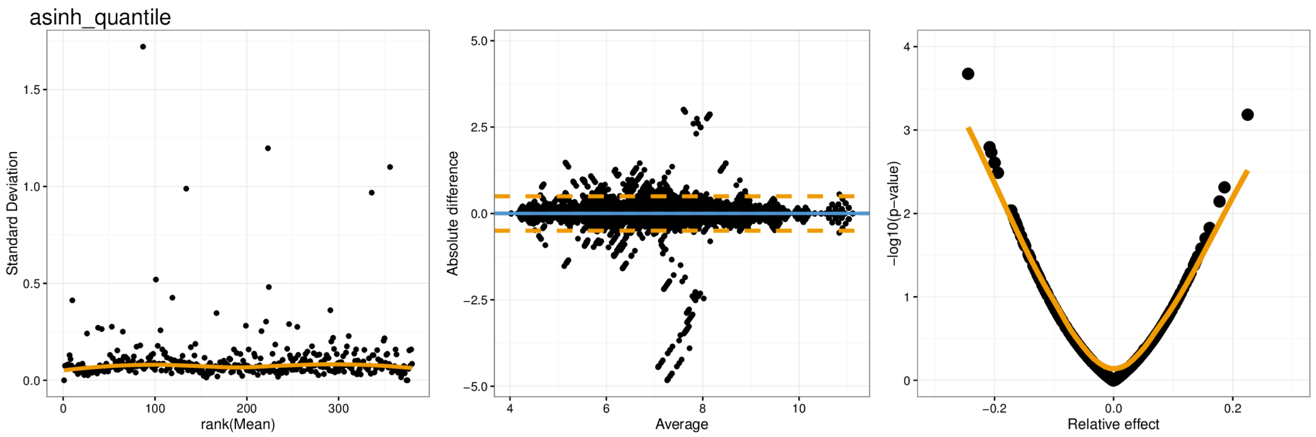

Figure S52: Mean-SD plot (left), Bland-Altman plot (middle), Volcano plot (right). Used method (top left) is displayed as *transformation_normalization*.

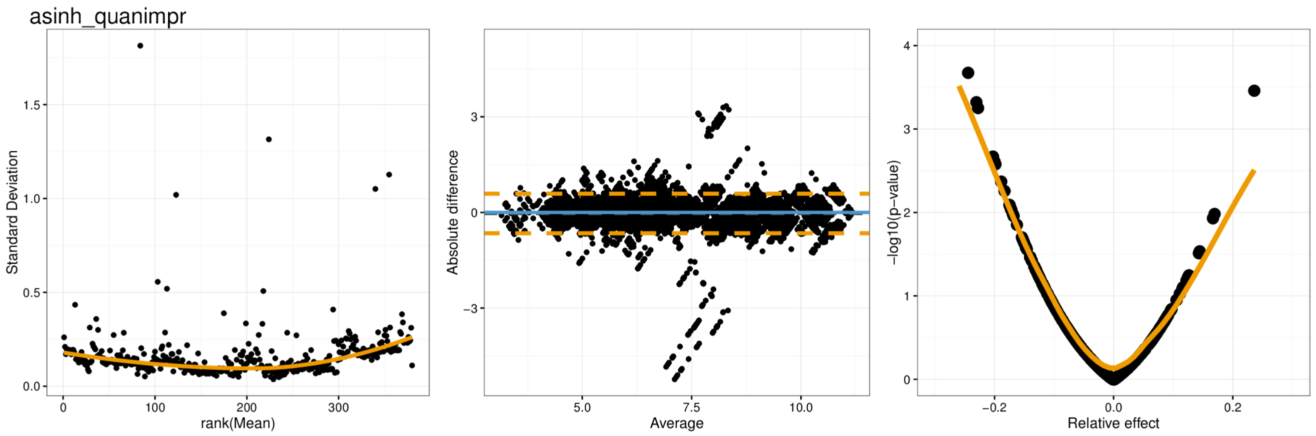

Figure S53: Mean-SD plot (left), Bland-Altman plot (middle), Volcano plot (right). Used method (top left) is displayed as *transformation_normalization*.

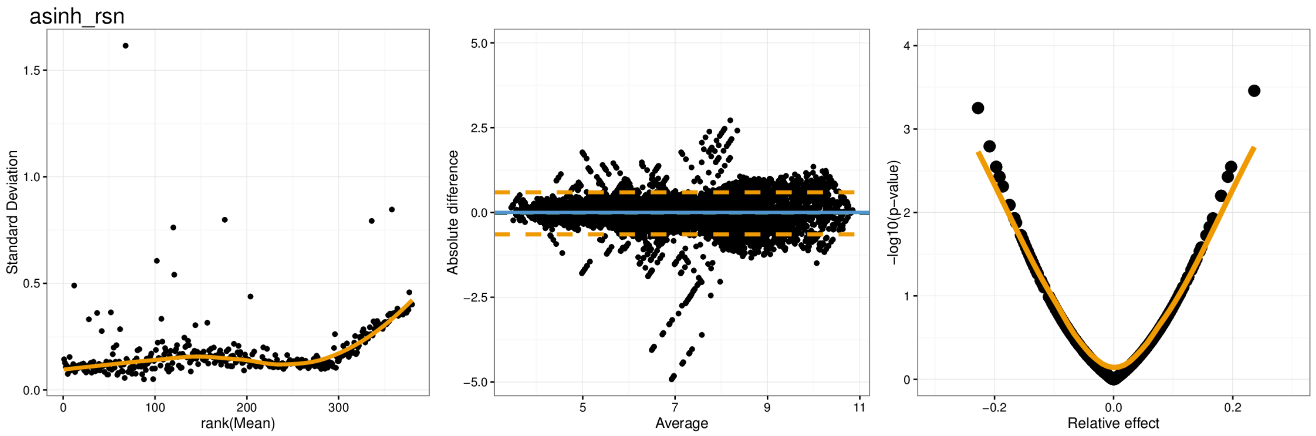

Figure S54: Mean-SD plot (left), Bland-Altman plot (middle), Volcano plot (right). Used method (top left) is displayed as *transformation_normalization*.

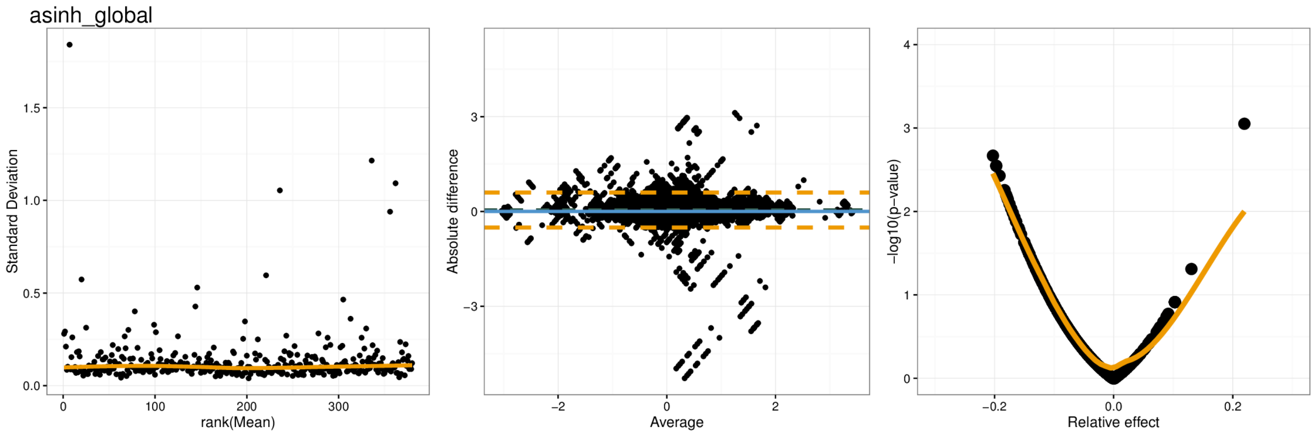

Figure S55: Mean-SD plot (left), Bland-Altman plot (middle), Volcano plot (right). Used method (top left) is displayed as *transformation_normalization*.

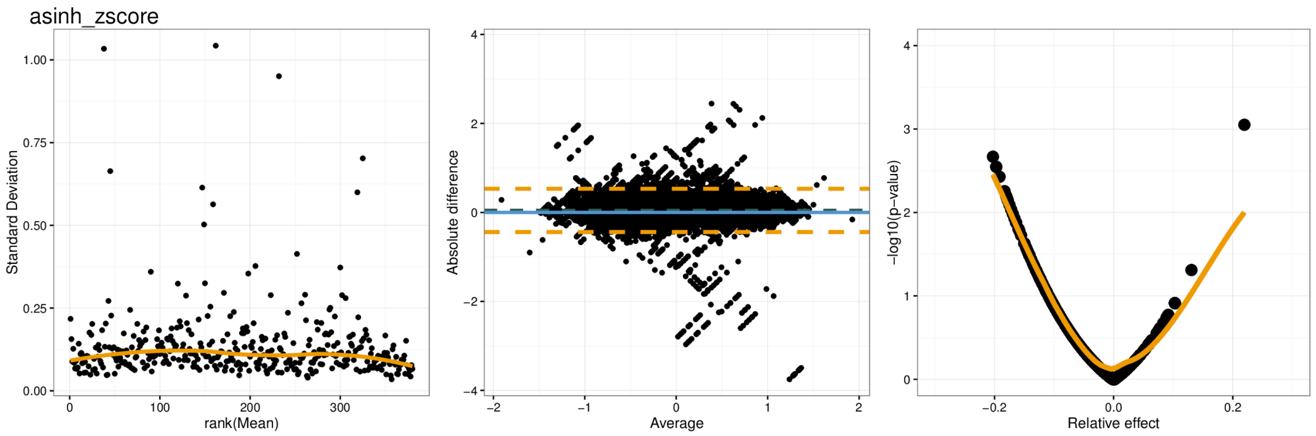

Figure S56: Mean-SD plot (left), Bland-Altman plot (middle), Volcano plot (right). Used method (top left) is displayed as *transformation_normalization*.

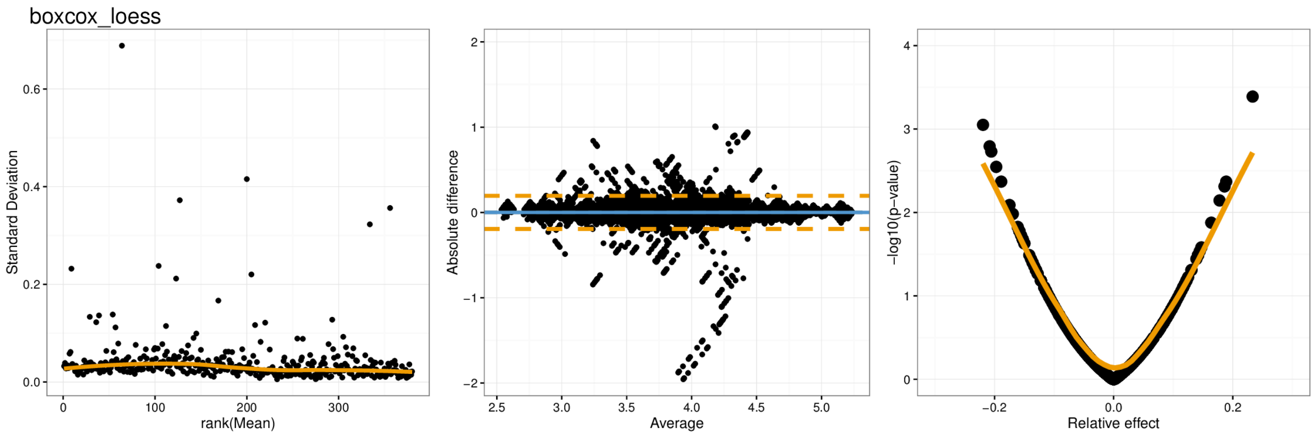

Figure S57: Mean-SD plot (left), Bland-Altman plot (middle), Volcano plot (right). Used method (top left) is displayed as *transformation_normalization*.

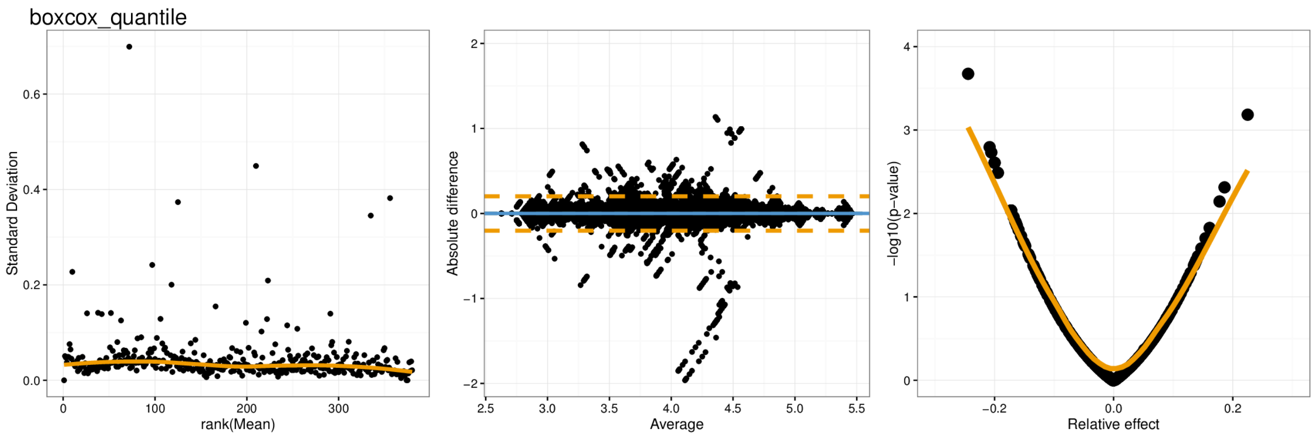

Figure S58: Mean-SD plot (left), Bland-Altman plot (middle), Volcano plot (right). Used method (top left) is displayed as *transformation_normalization*.

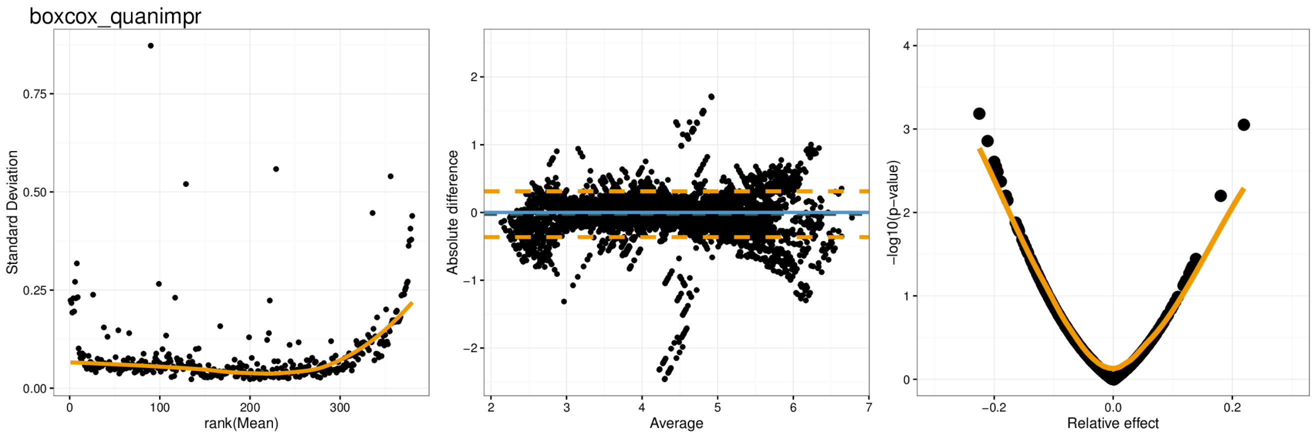

Figure S59: Mean-SD plot (left), Bland-Altman plot (middle), Volcano plot (right). Used method (top left) is displayed as *transformation_normalization*.

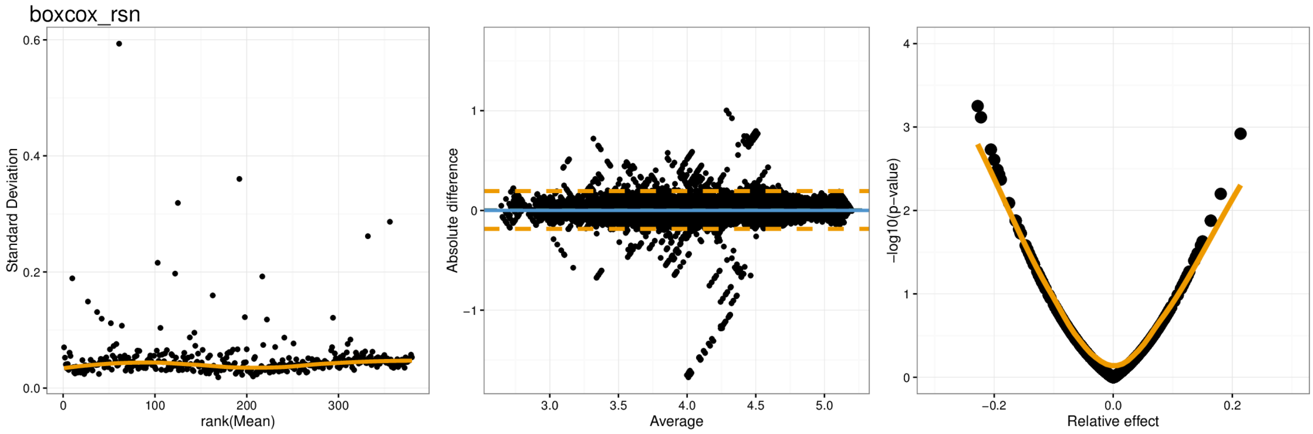

Figure S60: Mean-SD plot (left), Bland-Altman plot (middle), Volcano plot (right). Used method (top left) is displayed as *transformation_normalization*.

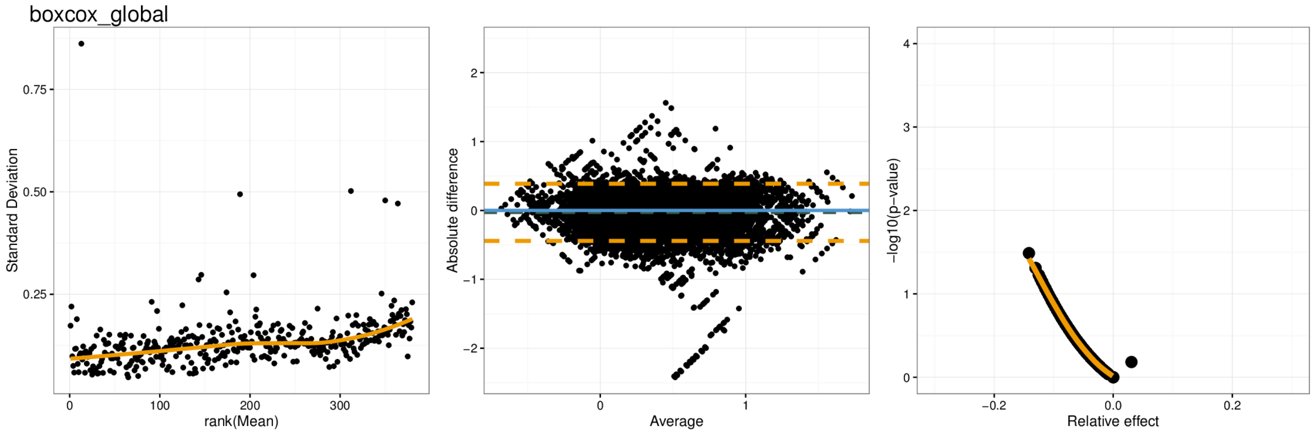

Figure S61: Mean-SD plot (left), Bland-Altman plot (middle), Volcano plot (right). Used method (top left) is displayed as *transformation_normalization*.

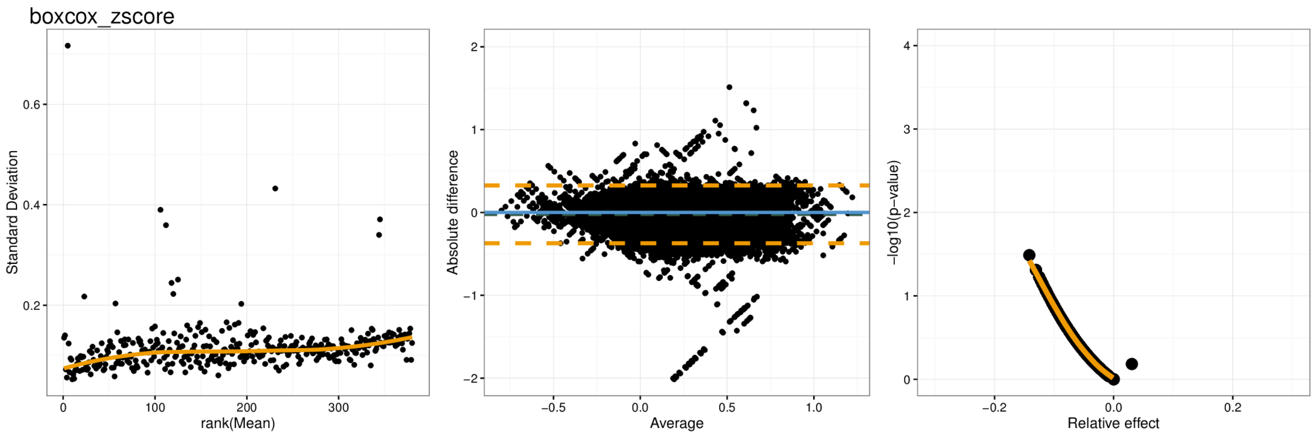

Figure S62: Mean-SD plot (left), Bland-Altman plot (middle), Volcano plot (right). Used method (top left) is displayed as *transformation_normalization*.

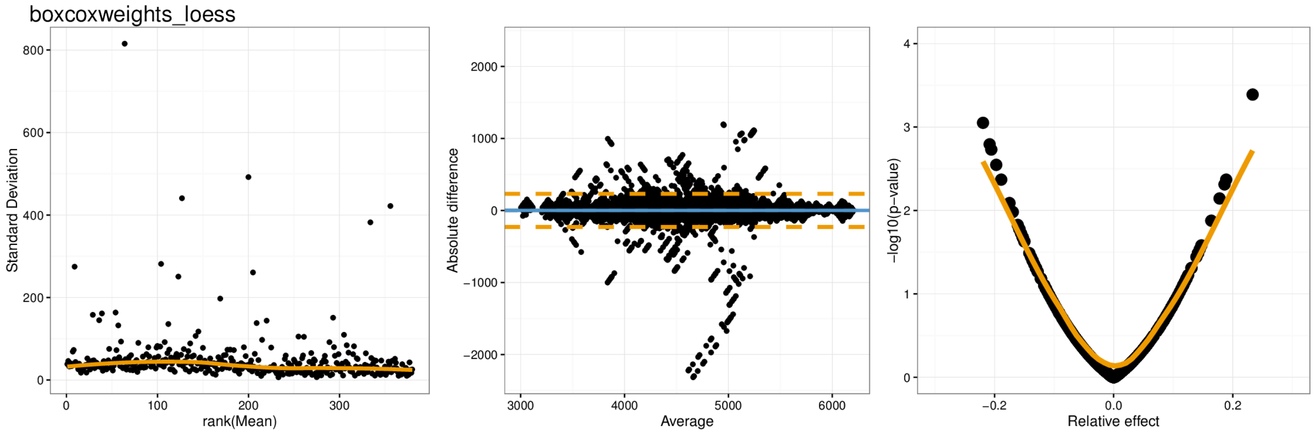

Figure S63: Mean-SD plot (left), Bland-Altman plot (middle), Volcano plot (right). Used method (top left) is displayed as *transformation_normalization*.

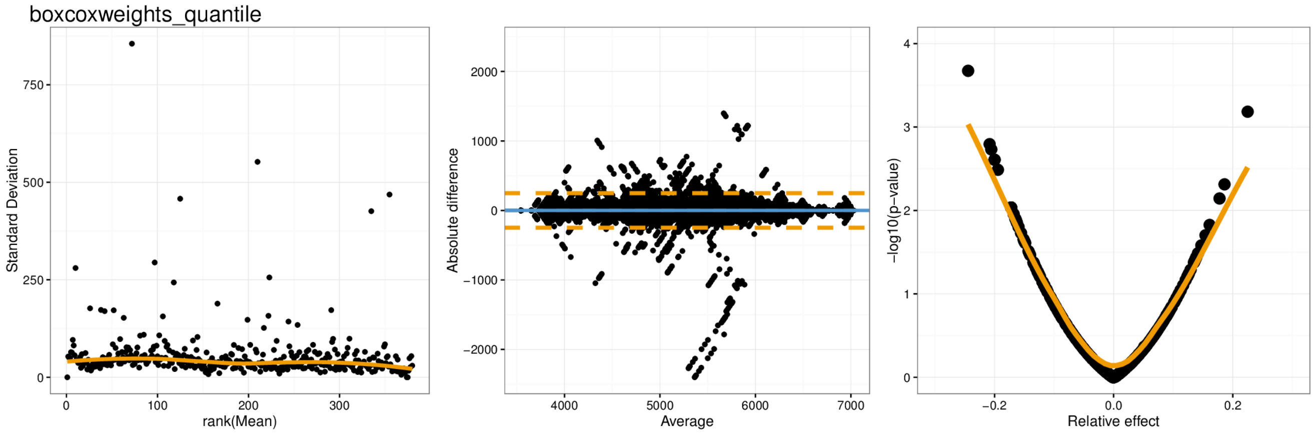

Figure S64: Mean-SD plot (left), Bland-Altman plot (middle), Volcano plot (right). Used method (top left) is displayed as *transformation_normalization*.

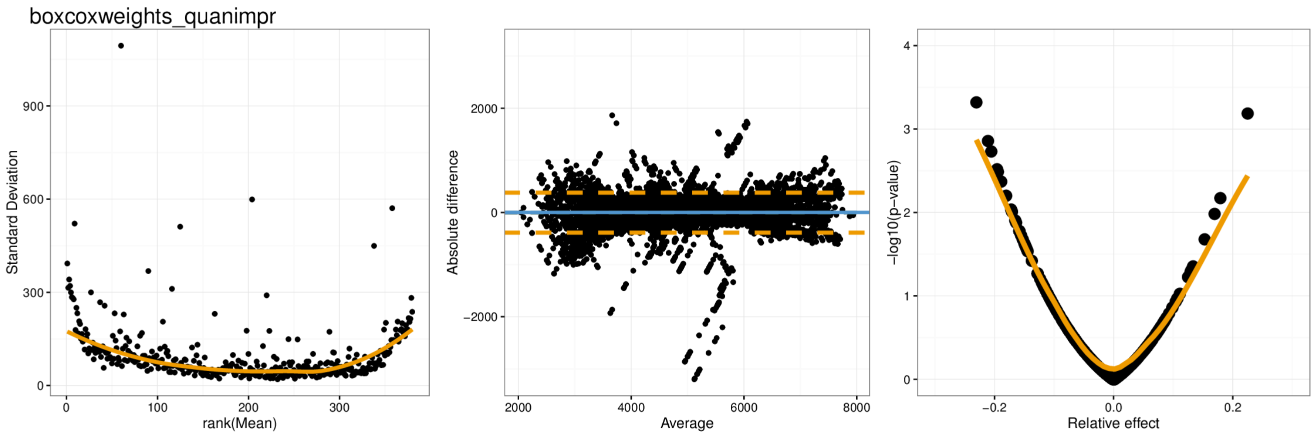

Figure S65: Mean-SD plot (left), Bland-Altman plot (middle), Volcano plot (right). Used method (top left) is displayed as *transformation_normalization*.

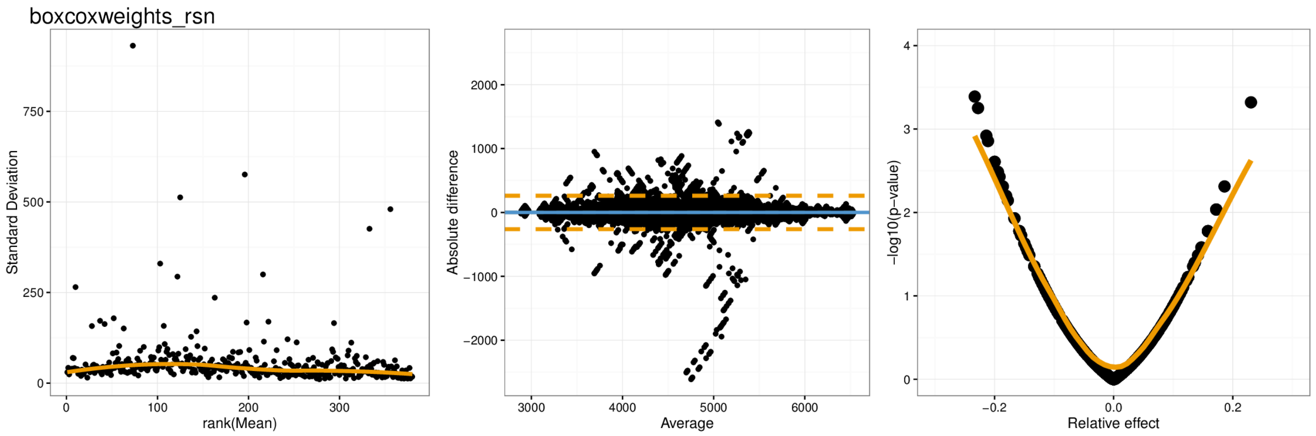

Figure S66: Mean-SD plot (left), Bland-Altman plot (middle), Volcano plot (right). Used method (top left) is displayed as *transformation_normalization*.

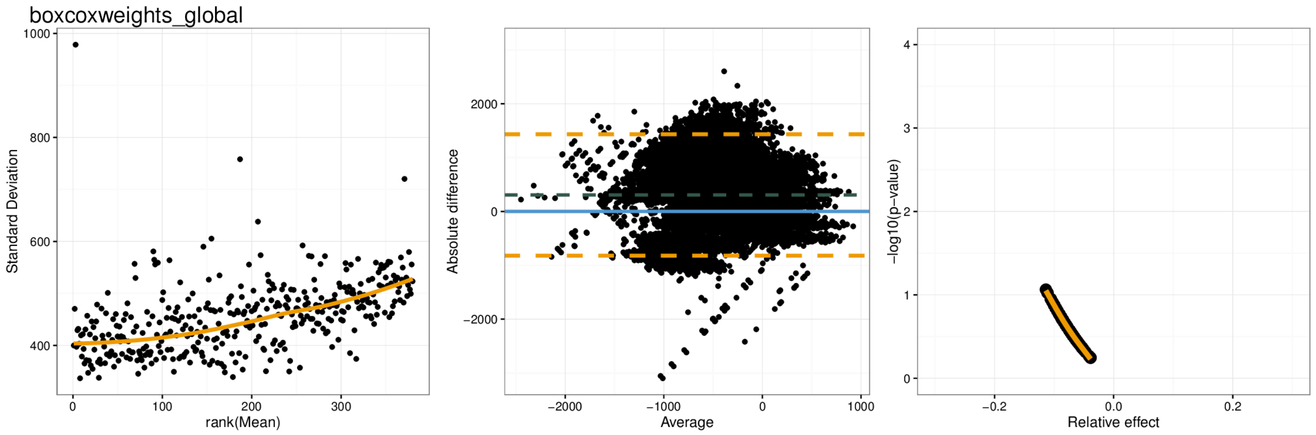

Figure S67: Mean-SD plot (left), Bland-Altman plot (middle), Volcano plot (right). Used method (top left) is displayed as *transformation_normalization*.

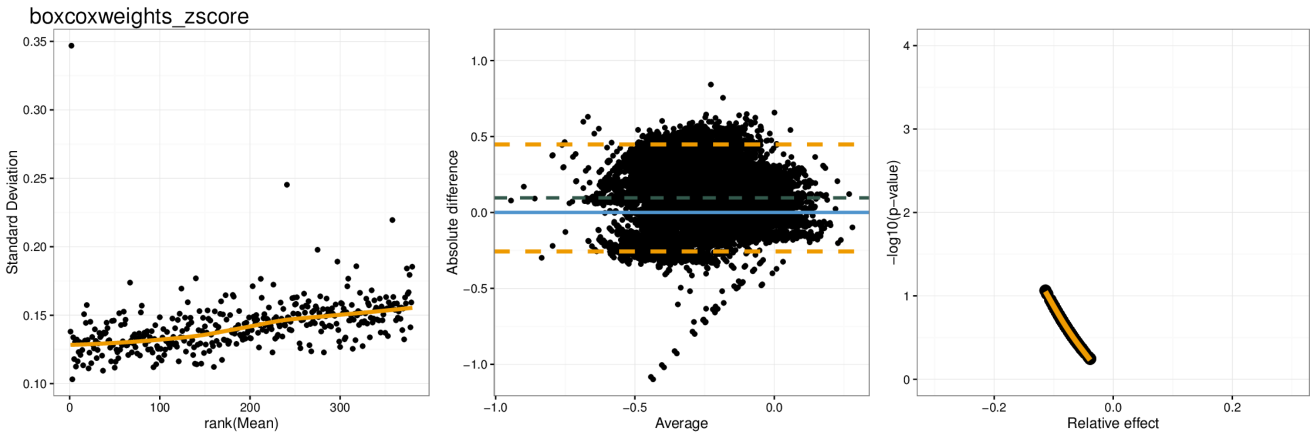

Figure S68: Mean-SD plot (left), Bland-Altman plot (middle), Volcano plot (right). Used method (top left) is displayed as *transformation_normalization*.

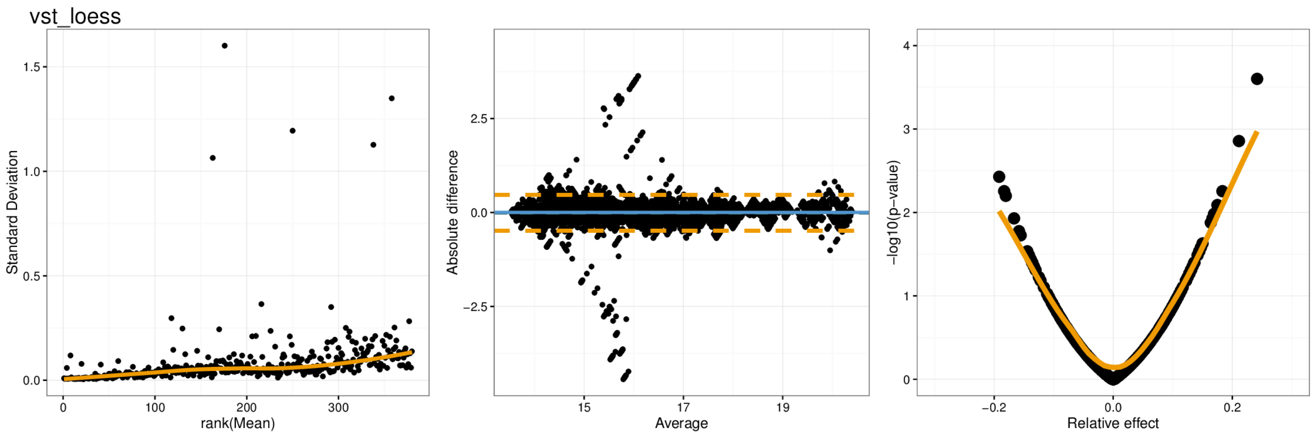

Figure S69: Mean-SD plot (left), Bland-Altman plot (middle), Volcano plot (right). Used method (top left) is displayed as *transformation_normalization*.

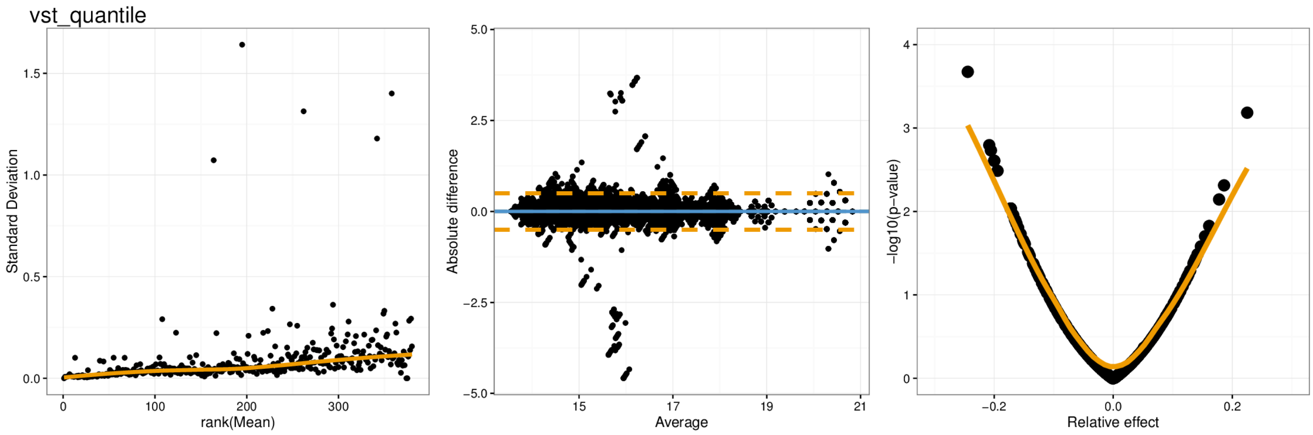

Figure S70: Mean-SD plot (left), Bland-Altman plot (middle), Volcano plot (right). Used method (top left) is displayed as *transformation_normalization*.

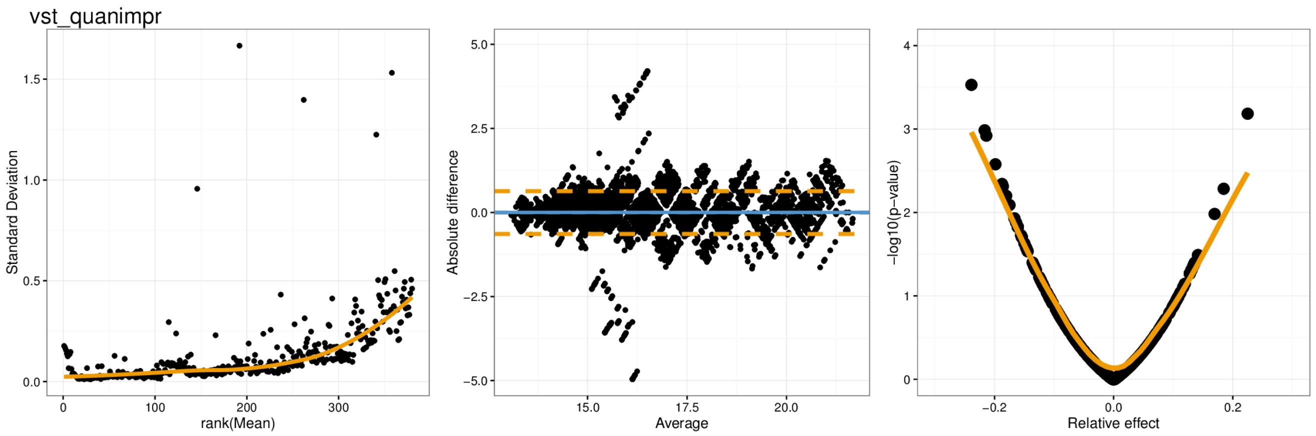

Figure S71: Mean-SD plot (left), Bland-Altman plot (middle), Volcano plot (right). Used method (top left) is displayed as *transformation_normalization*.

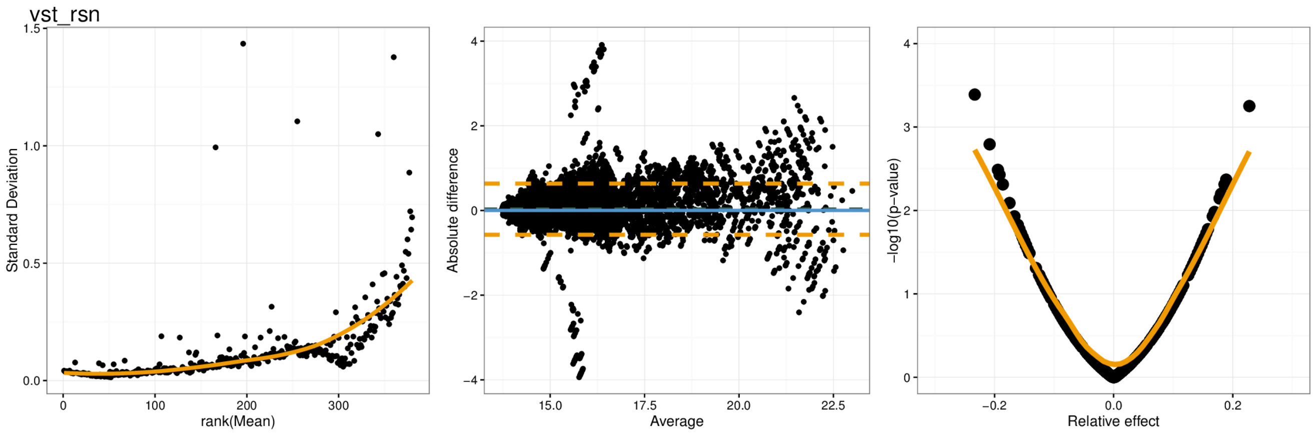

Figure S72: Mean-SD plot (left), Bland-Altman plot (middle), Volcano plot (right). Used method (top left) is displayed as *transformation_normalization*.

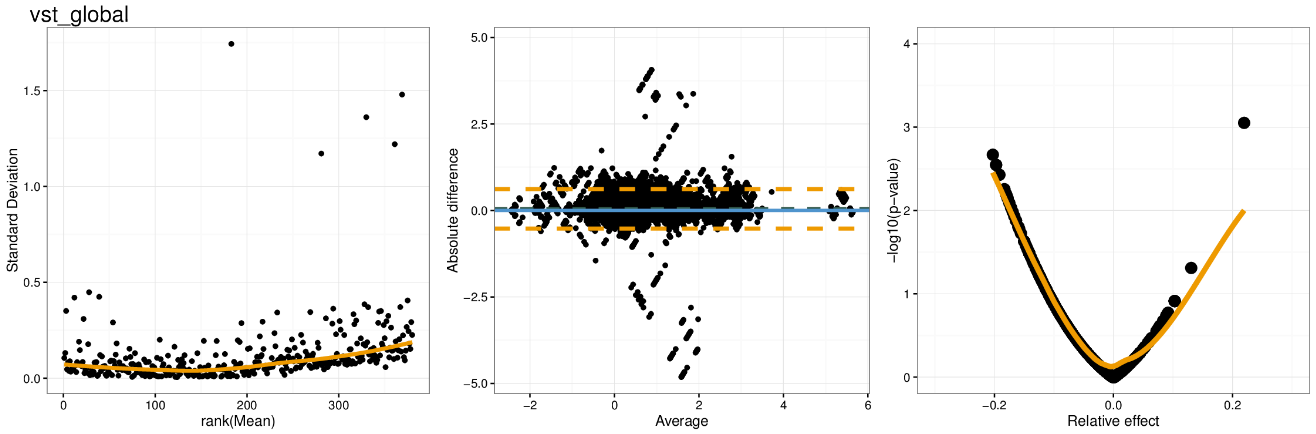

Figure S73: Mean-SD plot (left), Bland-Altman plot (middle), Volcano plot (right). Used method (top left) is displayed as *transformation_normalization*.

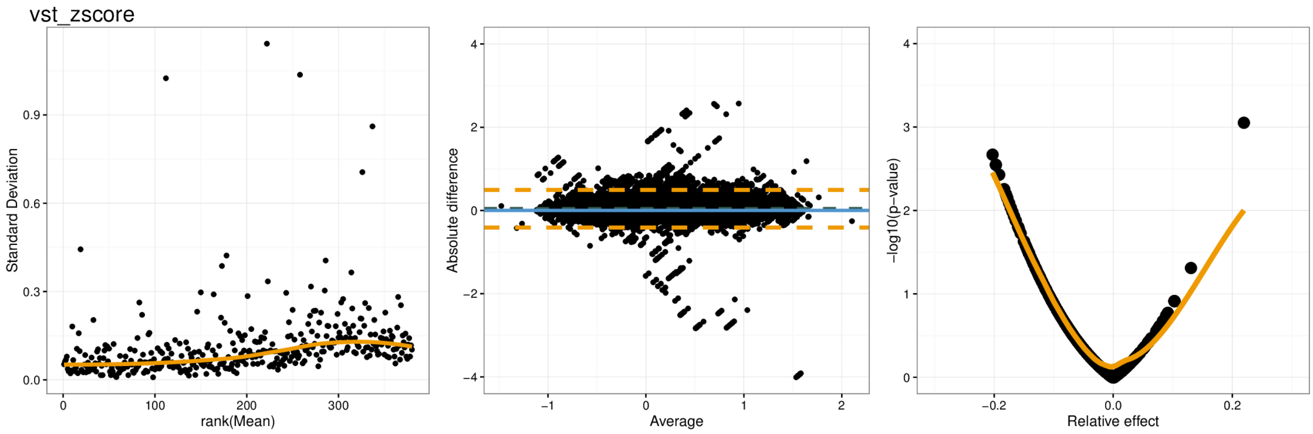

Figure S74: Mean-SD plot (left), Bland-Altman plot (middle), Volcano plot (right). Used method (top left) is displayed as *transformation_normalization*.

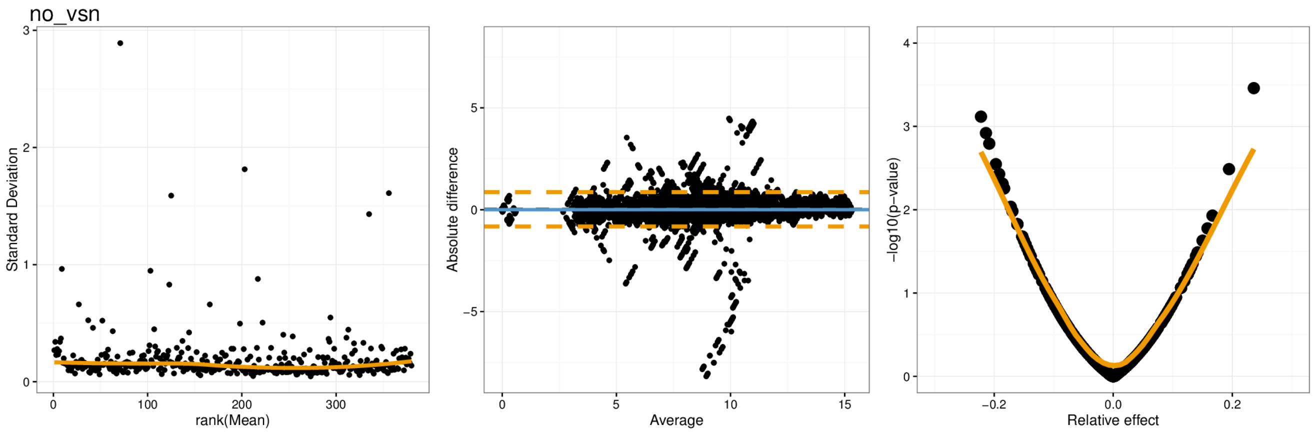

Figure S75: Mean-SD plot (left), Bland-Altman plot (middle), Volcano plot (right). Used method (top left) is displayed as *transformation_normalization*.
