# Supplementary material for: Comparison of pre-processing methods for multiplex bead-based immunoassays
Source: BMC Genomics. 2016 Aug 11;17:601. doi: 10.1186/s12864-016-2888-7 (PMC4982217; doi:10.1186/s12864-016-2888-7)
Supplement: Supplementary file 2 — Supplementary table. Detailed results for the evaluation criteria skewness, tail length and coefficient of variation. (PDF 54 kb) [file 12864_2016_2888_MOESM2_ESM.pdf]

Supplement for: *Comparison of Pre-processing Methods for Multiplex Bead-Based Immunoassays*

Tanja K. Rausch, Arne Schillert, Andreas Ziegler, Angelika Lueking, Hans-Dieter Zucht, Peter Schulz-Knappe

Part 4: Skewness, tail length and coefficient of variation for all 37 different pre-processing methods

Pre-processing method	Skewness	Tail length	Coefficient of variation
no_loess	3.3564	5.0443	79.9289
no_quantile	3.32	5.0093	80.0213
no_quanimpr	3.3521	5.2749	82.4751
no_rsn	2.054	3.2887	88.4993
no_global	1.4429	6.0856	55.1209
no_zscore	1.6482	3.3004	41.9559
log2_loess	0.4291	1.7434	54.8009
log2_quantile	0.4454	1.7552	55.9673
log2_quanimpr	0.3948	1.7798	67.596
log2_rsn	-0.1697	1.8272	71.2873
log2_global	0.1649	2.0798	48.7411
log2_zscore	0.3437	1.9315	46.6501
asinh_loess	0.429	1.7427	49.0967
asinh_quantile	0.4455	1.7552	50.3272
asinh_quanimpr	0.3942	1.7822	65.3201
asinh_rsn	-0.0781	1.7853	66.394
asinh_global	0.1649	2.0799	49.8506
asinh_zscore	0.3437	1.9315	49.3457
boxcox_loess	-0.0057	1.6672	34.0007
boxcox_quantile	0.0272	1.6856	37.2333
boxcox_quanimpr	0.3261	2.1103	56.6501
boxcox_rsn	-0.0144	1.6417	45.3983
boxcox_global	-0.0374	1.9627	78.5349
boxcox_zscore	0.0922	1.9263	74.9218
boxcoxweights_loess	-0.0057	1.6672	34.0789
boxcoxweights_quantile	0.0236	1.6852	33.0725
boxcoxweights_quanimpr	0.3303	2.4182	54.0541
boxcoxweights_rsn	0.0467	1.6436	40.4125
boxcoxweights_global	0.8775	2.8134	11.7994
boxcoxweights_zscore	0.9512	2.8565	11.6287
vst_loess	1.71	2.1547	26.2518
vst_quantile	1.7388	2.1068	26.394
vst_quanimpr	1.8008	2.0988	35.2347
vst_rsn	1.96	2.8408	35.6117
vst_global	0.5529	2.6842	52.6671
vst_zscore	1.0909	2.1658	45.6259
no_vsn	0.1951	1.851	63.0512

Table S1: Detailed results for evaluation criterias skewness, tail length and coefficient of variation for all 37 different pre-processing methods. Pre-processing methods are displayed as *transformation_normalization*.
